# Next-generation cell lines for profiling different proteasome forms and their implications in cancer

**DOI:** 10.3389/fimmu.2025.1672000

**Published:** 2025-12-16

**Authors:** Alexander Burov, Anastasia Yakovleva, Georgy Linovskiy, Andrei Fedotov, Vladimir Popenko, Olga Leonova, Aleksandr Lanin, Marina Drutskaya, Vadim Karpov, Alexey Morozov

**Affiliations:** 1 Laboratory of Regulation of Intracellular Proteolysis, Engelhardt Institute of Molecular Biology, Russian Academy of Sciences, Moscow, Russia; 2 Laboratory of Molecular Mechanisms of Immunity, Engelhardt Institute of Molecular Biology, Russian Academy of Sciences, Moscow, Russia; 3 Biology Department, M.V. Lomonosov Moscow State University, Moscow, Russia; 4 Center for Precision Genetic Technologies for Medicine, Engelhardt Institute of Molecular Biology, Russian Academy of Sciences, Moscow, Russia; 5 Physics Department, M.V. Lomonosov Moscow State University, Moscow, Russia; 6 Life Improvement by Future Technologies (LIFT) Center, Moscow, Russia; 7 Department of Cancer Cell Biology, Engelhardt Institute of Molecular Biology, Russian Academy of Sciences, Moscow, Russia

**Keywords:** proteasome, constitutive proteasome, immunoproteasome, intermediate proteasome, reporter cell line

## Abstract

**Background:**

Most intracellular proteins undergo degradation by proteasomes, which exist in constitutive, immune, and intermediate forms. The diversity arises from the presence of different catalytic β subunits incorporated into the 20S core particle. Constitutive proteasomes contain β1, β2 and β5 subunits, whereas immunoproteasomes integrate β1i, β2i and β5i proteins. Intermediate proteasomes comprise combinations of constitutive and immune subunits, but typically lack the β2i subunit. Distinct proteasome configurations have widespread impact on cellular metabolism, gene expression, immune signaling, stress adaptation and tumorigenesis. However, the biological functions of proteasome subtypes remain incompletely defined, underscoring the need for refined experimental systems to uncover their specific activities.

**Methods:**

Previously, we generated cancer cell lines expressing mCherry-tagged β5i subunit under the control of endogenous regulatory elements. Using CRISPR/Cas9 nickase technology, we further modified the genomes of the colon adenocarcinoma SW620B8-mCherry and cervical adenocarcinoma TZM-blB8-mCherry cells. In SW620B8-mCherryB5-GFP cells, the *eGFP* gene was inserted at the 3’ end of the *PSMB5* gene, which encodes the β5 subunit. Similarly, the β2i-encoding gene (*PSMB10*) was fused with the photo-switchable cyan fluorescent protein gene (*PS-CFP2*) in TZM-blB8-mCherryB10-CFP cells.

**Results:**

Efficient integration of fluorescent protein-encoding sequences into target genomes provided robust expression and integration of chimeric subunits into proteasomes. In TZM-blB8-mCherryB10-CFP cells, photo-conversion of PS-CFP2 allowed visualization of both intermediate and immunoproteasomes within the cellular nuclei following treatment with IFN-γ/TNF. In SW620B8-mCherryB5-GFP cells, analysis of intracellular proteasome localization revealed discrete regions enriched in β5i-containing proteasomes. Moreover, IFN-γ/TNF exposure induced a fluorescence shift from green to red, accompanied by redistribution of intracellular proteasomes in SW620B8-mCherryB5-GFP cells. Multiphoton microscopy of tumors grafted into immunocompromised mice showed the formation of β5i-containing, proteasome-enriched inclusions and suggested their potential release from cancer cells. Collectively, the TZM-blB8-mCherryB10-CFP cell line provides a tool to interrogate immunoproteasome functions independently of intermediate proteasomes, whereas SW620B8-mCherryB5-GFP cells enable visual discrimination between constitutive and β5i-containing proteasomes both *in vitro* and *in vivo*.

**Conclusions:**

Generated cell lines provide a novel platform for dissecting proteasome functions, addressing the distinct properties of individual proteasome forms, and assessing the impact of diverse compounds on the proteasome repertoire in cultured cells and in tumor xenografts.

## 1 Introduction

Antigen presentation relies on the demonstration of peptides, derived from self or foreign proteins, to T lymphocytes. Most intracellular proteins are processed into peptides by the ubiquitin-proteasome system (UPS), which includes enzymes that conjugate ubiquitin to selected substrates and direct their subsequent degradation (1). The hydrolysis of ubiquitinated proteins is mediated by proteasomes – large (700–2500 kDa) multisubunit proteases. The 20S proteasome core particle consists of four heptameric layers of alpha and beta subunits arranged in an alpha(1-7)/beta(1-7)/beta(1-7)/alpha(1-7) configuration (2). Proteolytic activity is associated with three beta subunits that confer caspase-like (β1), trypsin-like (β2) and chymotrypsin-like (β5) specificities. The standard β1, β2, and β5 subunits are integrated into constitutive proteasomes (cPs), which represent the predominant proteasome form in most cells types of the organism (**Table 1**). However, during proteasome assembly, constitutive subunits can be substituted by their immune analogs β1i, β2i and β5i (19, 20). Proteasomes incorporating the immune subunits exhibit reduced caspase-like activity but elevated chymotrypsin-like activity, thereby generating peptides enriched in hydrophobic amino acids at the C-terminus. This structural feature is essential for efficient loading of peptides onto MHC class I molecules and their presentation on the cell surface. In line with this, immunoproteasomes (iPs) are highly abundant in antigen-presenting cells and other immune lineages (**Table 1**). At the same time, immunoproteasomes are also induced in a broad range of non-immune cells under stress conditions or upon exposure to pro-inflammatory cytokines such as IFN-γ and TNF (12, 14). Notably, immune subunits do not necessarily replace all of the constitutive analogues within the proteasome (7). Under physiological conditions, proteasomes containing combinations of immune and constitutive β subunits are abundant in the liver, kidney, and dendritic cells (**Table 1**) (5). These intermediate proteasomes (intPs) commonly incorporate the β5i, while lacking the β2i subunit (5). The overview of known proteasome forms can be found in (10, 21, 22). Various proteasome subtypes generate altering sets of peptides and demonstrate different capacity towards oxidized and damaged proteins (8, 23–26). Collectively, current data support the concept of proteasome heterogeneity reflecting cellular state, adaptation to different stimuli, functional specialization and systemic effects.

**Table 1:** Major known proteasome forms. *Tissue specific proteasome forms are not included.

Proteasome form	Catalytic subunit composition	Tissue distribution	References
Constitutive proteasome (cP) 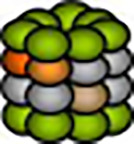	2β1, 2β2, 2β5	Highly expressed in different tissues, including the brain, lung, heart, kidney and skeletal muscles. Among cell types with high expression are erythrocytes, as well as embryonic and cancer cell lines.	(3–11)
Immunoproteasome (iP) 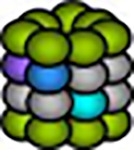	2β1i, 2β2i, 2β5i	Abundant in spleen, low levels were observed in the liver, kidney, heart. Up to 64% of the proteasome pool is represented by iPs in the the small intestine and colon mucosa. The iPs are expressed in T- and B-lymphocytes, NK-cells, monocytes, as well as dendritic cells. The iPs were also identified in the medullary thymic epithelial cells. Various cells express iPs under stress conditions and following stimulation with pro-inflammatory cytokines.	(3, 5, 8, 9, 11–17)
Intermediate proteasome (intP) β5i 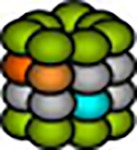	2β1, 2β2, 2β5i	Represent one half of the proteasome pool in liver and 30% of the proteasome pool in the kidney. Found in small intestine and colon mucosa. IntPs are abundant in immature and mature dendritic cells and represent the major proteasome form in cells of the U937 histiocytic lymphoma cell line.	(3, 5, 18)
β1i-β5i 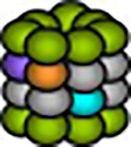	2β1i, 2β2, 2β5i.	Constitute up to 54% of the proteasome pool in monocytes. Represent a significant portion of the proteasome pool within cells of an acute myelogenous leukemia cell line KG1a.	(3, 5)

The distinct functions of different proteasome forms are highlighted by their up- or downregulation in the case of severe pathologies, including cancer, autoimmune and neurodegenerative diseases (27–29). Consequently, targeted modulation of specific proteasome subtypes by altering their expression or by modifying their activity is considered as a promising therapeutic strategy. For example, a recently identified compound stimulates β5i expression, expands the immunopeptidome of multiple myeloma cells, and delays tumor progression in animals injected with pretreated cancer cells (30). Conversely, an inhibitor selective for iP subunits has recently been tested in clinical trials, and numerous additional candidate molecules are currently under investigation (31). Nevertheless, understanding of the specific functions and “necessity” of each proteasome form remains incomplete. Although certain progress has been achieved in defining the roles of constitutive and immunoproteasomes, far less is known about the intermediate proteasomes. Their research is limited due to the difficulty in distinguishing between intPs and iPs using conventional experimental approaches. Thus, improved tools are required to address the contributions of each proteasome subtype and to facilitate the development of refined therapeutic strategies for diverse diseases, including cancer.

Direct visualization of proteasomes can help to overcome this challenge (32, 33). It should be mentioned that until recently, visualization of proteasomes in cells has primarily relied on immunofluorescence or transient transfection with plasmids encoding fluorescently tagged proteasome subunits. In addition, lentiviral transduction has been used to generate stable cell lines expressing such chimeric subunits. Although these approaches have enabled important insights into proteasome biology, they possess significant limitations. Specifically, the genomic integration site of the transgene is unpredictable, meaning that expression of the chimeric subunit is not controlled by the endogenous regulatory elements that normally govern proteasome subunit expression. Therefore, these models are poorly suited for studying stimulus-induced changes in proteasome subunit expression, including those driven by cytokines or bioactive compounds. Moreover, overexpression of chimeric subunits can disrupt the tightly regulated balance of proteasome subunit synthesis and the complex assembly. Furthermore, not all of the overexpressed subunits will be integrated into proteasomes. This complicates accurate interpretation of proteasome localization and dynamics. In this context, CRISPR/Cas9-based genome editing represents a superior molecular tool for improving the generation of reporter cell lines through precise insertion of the reporter into endogenous loci, thereby preserving physiological regulation. Previously, using targeted genome editing, we established a panel of five cancer cell lines expressing the β5i subunit fused to the fluorescent protein mCherry under the control of endogenous regulatory elements (34, 35). The chimeric subunit was successfully incorporated into proteasomes, and its functionality was confirmed. However, these cell lines did not allow visualization of constitutive proteasomes or enable discrimination between intPs and iPs.

Here, using a CRISPR/Cas9D10A nickase, we further modified two of the previously engineered cell lines in order to create molecular tools for simultaneous visualization of constitutive and β5i-contaning proteasomes and elucidation of the specific functions of iPs and intPs.

## 2 Materials and methods

### 2.1 Mice

The NSG-SGM3 mice (NOD.Cg-Prkdcscid Il2rgtm1Wjl Tg(CMV-IL3,CSF2,KITLG) 1Eav/MLoySzJ, JAX stock #013062) were obtained from the Jackson Laboratory (Bar Harbor, ME, USA) and were kindly shared by Prof. O. Demidov as part of the RSF project 19-75-20128. This strain was originally described in (36 and 37) and was maintained at the Animal Facility of the Center for Precision Genetic Technologies for Medicine, EIMB RAS. For anesthesia, a mixture of 5% Zoletil 100 (Virbac, France) and 15% Xylazine (Interchemie, Netherlands) in saline was used; 100 μl of the solution was injected intraperitoneally per 20 g mouse. Euthanasia was performed by administering a threefold overdose of the anesthetic to preserve tissues for further perfusion. All animal procedures were performed in accordance with Russian regulations on animal protection and were approved by the local Ethics Review Committee of EIMB RAS (Protocol No. 1 from March the 5th 2025).

### 2.2 Cell lines

Human colorectal adenocarcinoma cell lines SW620 and SW620B8-mCherry and cervical adenocarcinoma cells TZM-bl and TZM-blB8-mCherry were obtained and characterized previously (34). SW620, TZM-bl, SW620B8-mCherry, TZM-blB8-mCherry, SW620B8-mCherryB5-GFP and TZM-blB8-mCherryB10-CFP cells were cultured in Dulbecco’s Modified Eagle Medium (DMEM) (PanEko, Moscow, Russia), or phenol red-free DMEM (Thermo Fisher Scientific, Waltham, MA, USA) supplemented with 10% fetal calf serum (FCS) (Hyclone, Logan, UT, USA), penicillin (100 U/mL), and 100 μg/mL streptomycin (PanEko, Moscow, Russia), at 37^0^ C and 5% CO_2_.

### 2.3 Molecular cloning

Gene knock-in was performed using the CRISPR/Cas9D10A nickase. To modify the genomes of SW620B8-mCherry and TZM-blB8-mCherry cell lines, two vectors were designed for each genome editing. The first vector in both cases was based on the pDG461 plasmid (AddGene, Watertown, MA, USA) and contained a gene encoding Cas9(D10A), fused via a T2A sequence to an *eGFP*, as well as two specific gRNAs (**Supplementary Table 1**). Pairs of gRNAs were engineered to introduce nicks on the sides of the *PSMB5* and *PSMB10* stop codons, positioned 59 bp and 60 bp apart, respectively. All gRNA sequences were selected through bioinformatic analysis (COSMID ^1^and Cas-OFFinder software^2^) to minimize potential off-target cleavage.

**Figure 1:**
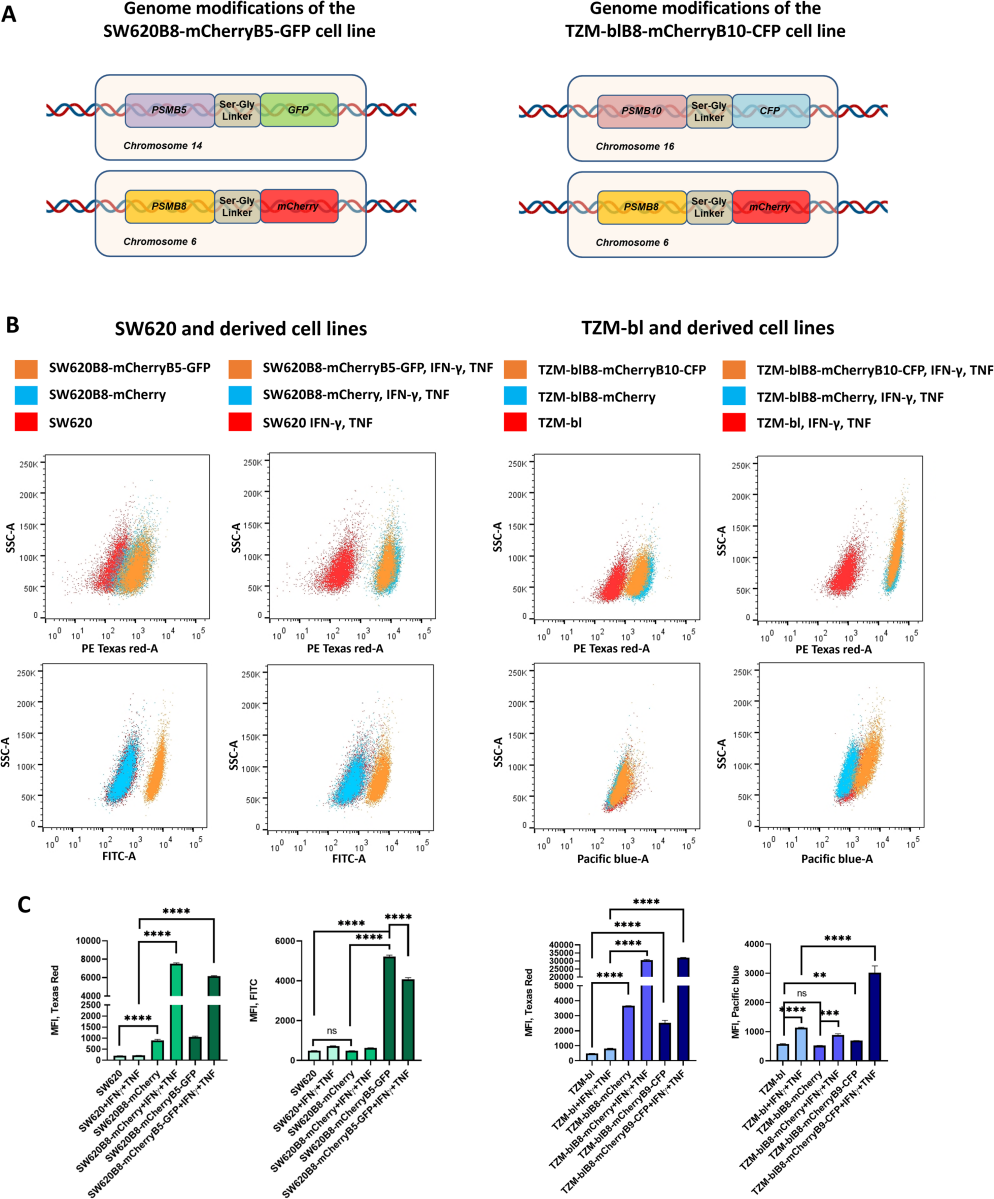
Generation of SW620B8-mCherryB5-GFP and TZM-blB8-mCherryB10-CFP cell lines. **(A)** Schematic representation of genome modifications in SW620B8-mCherryB5-GFP (left) and TZM-blB8-mCherryB10-CFP (right) cell lines. **(B)** Analysis of SW620- and TZM-bl-derived cells by flow cytometry. Populations of control cells and cells stimulated for 72 h with pro-inflammatory cytokines (1000 U/mL of recombinant human IFN-γ and 500 U/mL of recombinant human TNF) are shown. Control cell populations are shown in red; cells expressing one chimeric subunit – in blue, and cells with two chimeric subunits – in orange. For each cell population, FSC vs SSC gating was used to exclude dead cells and cell debris; FSC area vs height gating was done to exclude cell doublets. 10.000 events are shown in each case. **(C)** Mean fluorescence intensity of mCherry, EGFP and PS-CFP2 in SW620 (left) and TZM-bl (right) -derived cells and the same cell lines treated with 1000 U/mL of recombinant human IFN-γ and 500 U/mL of recombinant human TNF. Tests were performed in triplicate. **p<0.01; ***p<0.001; ****p<0.0001; t-test.

The second set of vectors contained the donor sequences for homologous recombination. These constructs were obtained using pAL-2T plasmid (Evrogen, Moscow, Russia) as a backbone. A fluorescent protein gene (*eGFP* to fuse with the *PSMB5* or *PS-CFP2* (Evrogen, Moscow, Russia) to be fused with the *PSMB10* (**Figure 1A**), Ser-Gly (GSGGGGSGGGGSGT) linker-encoding sequence, as well as homology arms carrying PAM-blocking mutations, were cloned into the pAL-2T using the NEBuilder HiFi DNA Assembly Kit (New England Biolabs, Ipswich, MA, USA). The resulting vectors were amplified in *E.coli* (New England Biolabs, Ipswich, MA, USA). Plasmids were purified using the Plasmid Miniprep Color kit (Evrogen, Moscow, Russia), according to the manufacturer’s protocol. Plasmid DNA concentration and quality were evaluated using the NanoDrop spectrophotometer (Thermo Fisher Scientific, Waltham, MA, USA). Bi-directional sequencing was performed to validate the correct sequence of the inserts.

### 2.4 Transfection and cell sorting

The SW620B8-mCherry and TZM-blB8-mCherry cells were co-transfected with the obtained plasmids using TransIT-LT1 transfection reagent (Mirus Bio LLC, Madison, WI, USA), according to the manufacturer’s instructions. Forty-eight hours after the transfection cells were washed twice with PBS and detached from the culture plate using trypsin-EDTA solution (PanEko, Moscow, Russia). The cells were centrifuged at 200 x g for 5 min and the cellular pellets were resuspended in 1 mL of PBS containing 2% FCS. Then, cells with green fluorescence were sorted using the BD FACSAria III cell sorter (BD Biosciences, Franklin Lakes, NJ, USA). Two weeks later, SW620B8-mCherryB5-GFP cells were sorted again to enrich for cells with bright GFP fluorescence. Cells with PS-CFP2 fluorescence were sorted from the TZM-blB8-mCherryB10-CFP cells following 72 h stimulation with 1000 U/mL of recombinant human IFN-γ and 500 U/mL of recombinant human TNF (both from R&D systems, Minneapolis, MN, USA). From two (SW620B8-mCherryB5-GFP) to five (TZM-blB8-mCherryB10-CFP) additional rounds of sorting or sorting preceded by cytokine stimulation were performed to obtain populations consisting of fluorescent and inducible cells (**Figure 1B**, **Supplementary Figure 1**).

### 2.5 Isolation of genomic DNA and RNA, PCR and cDNA synthesis

Genomic DNA was isolated using the ExtractDNA Blood and cells kit (Evrogen, Moscow, Russia), according to the manufacturer’s instructions. The concentration and purity of nucleic acids were evaluated using a NanoDrop spectrophotometer (Thermo Fisher Scientific, Waltham, MA, USA). Successful incorporation of genes into target cell genomes was confirmed using PCR with primer pairs designed in a way that one primer is located within the coding sequence of the inserted gene, and the other - within the cellular genome (**Supplementary Table 1**; **Supplementary Figure 2**).

Total RNA was extracted using the RNA Solo kit (Evrogen, Russia). The RapidOut DNA Removal kit (Thermo Fisher Scientific, Waltham, MA, USA) was used to remove DNA from the obtained RNA samples. cDNA was synthesized by reverse transcription using an oligo(dT)18 primer and the Magnus Reverse Transcriptase (Evrogen, Moscow, Russia), according to the manufacturer’s protocol.

### 2.6 Real-time PCR

The real-time PCR was used to evaluate the expression levels of endogenous and chimeric genes. The expression of *PSMB5* and *PSMB8*, as well as of *PSMB5-eGFP* and *PSMB8-mCherry*, was studied in SW620 and SW620B8-mCherryB5-eGFP cells, respectively. mRNA was obtained from control cells and cells that were incubated with recombinant human IFN-γ (1000 mg/mL)/TNF (500 mg/mL) (both R&D Systems, Minneapolis, MN, USA) for 72 h. Previously designed primers were used to amplify endogenous genes fragments (38), whereas primer pairs “M”, “N” (*PSMB5-eGFP*) and “W”, “X” (*PSMB8-mCherry*) were used to evaluate chimeric genes expression (**Supplementary Table 1**; **Supplementary Figure 3**). Preservation of endogenous regulatory mechanisms that control *PSMB10-PS-CFP2* and *PSMB8-mCherry* expression in TZM-blB8-mCherryB10-PS-CFP2 was tested using qPCR after treatment of wt TZM-bl and modified cells with recombinant human IFN-γ (1000 mg/mL) and recombinant human TNF (500 mg/mL) (both R&D Systems, Minneapolis, MN, USA) for 72 h. Two sets of primers “O”,”P” (*PSMB10-PS-CFP2*) and “W”,”X”(*PSMB8-mCherry*), directed at chimeric genes, were used (**Supplementary Table 1**; **Supplementary Figure 3**). Expression of endogenous *PSMB8* and *PSMB10* was evaluated in untreated and cytokine-stimulated TZM-bl cells using primer pairs “U”,”V” and “S”,”T”, correspondingly (**Supplementary Table 1**; **Supplementary Figure 3**). The β-actin gene (*ACTB*) expression was used for normalization. Relative expression levels were calculated using the ΔΔCt method.

All primers were selected using the IDT software^3^ and the OligoAnalizer tool, following previously selected criteria (39).

### 2.7 Preparation of cellular lysates and Western blotting

Cells were washed twice with PBS and detached from the culture plate using a cell scraper (TPP, Trasadingen, Switzerland). After 5 min of centrifugation at 250 x g and 4^0^C cellular pellets were lysed in the ice-cold NP-40 cell lysis buffer (50 mM Tris-НCl (pH 8.0), 150 mM NaCl, 1.0% NP-40) for 10 min. The samples were then centrifuged for 20 min at 16.000 x g. Clarified homogenates were collected and stored at -80^0^C. The obtained homogenates were mixed with the SDS-PAGE Sample buffer (Thermo Fisher Scientific, Waltham, MA, USA), boiled for 10 min at 95^0^C and loaded onto a 12% Tris-glycine polyacrylamide SDS gel. Following electrophoresis, a wet transfer module (Bio-Rad, Hercules, CA, USA) was used to transfer proteins onto the nitrocellulose membranes (Bio-Rad, Hercules, CA, USA). Membranes were blocked in 6% skim milk (BioFroxx, Einhausen, Germany) prepared in PBS containing 0.1% Tween-20 (Thermo Fisher Scientific, Waltham, MA, USA). Blocked membranes were incubated with primary antibodies (**Supplementary Table 2**), washed five times with PBS-Tween, and then incubated with appropriate HRP-conjugated secondary antibodies (**Supplementary Table 2**). After that, blots were washed and developed using SuperSignal West Femto Maximum Sensitivity Substrate (Thermo Fisher Scientific, Waltham, MA, USA). To verify equal protein loading, membranes were stripped and re-stained with anti-β-actin primary antibodies (**Supplementary Table 2**) and relevant secondary antibodies (**Supplementary Table 2**).

### 2.8 Immunoprecipitation of proteasomes

The Proteasome Purification kit (Enzo, Farmingdale, NY, USA) was used for proteasome immunoprecipitation. Briefly, cells were homogenized by 5 cycles of freezing and thawing in a proteasome homogenization buffer (25 mM HEPES, pH 7.4, 10% glycerol, 5 mM MgCl_2_, 1 mM ATP, 1 mM DTT). Then homogenates were incubated for 10 min on ice and clarified by centrifugation at 13.000 x g for 30 min. Collected supernatants were mixed with the proteasome purification matrix and left overnight at 4^0^C with rotation. The following day, unbound fractions (Unb.) were collected after short (30s) centrifugation at 5000 x g. The slurry-containing pellets were washed 5 times with the homogenization buffer. To elute the proteasomes, the SDS-PAGE Sample buffer (Thermo Fisher Scientific, Waltham, MA, USA) was added to the pellets. Samples were heated to 95^0^C and analyzed by Western blotting.

Alternatively, cells were homogenized in buffer 1 (25 mM Tris-HCl, pH 7.4, 5 mM MgCl_2_, 1 mM ATP), incubated on ice and centrifuged as described above. Collected supernatants were incubated overnight at 4^0^C with anti-mCherry antibodies (Abcam, Cambridge, UK) (**Supplementary Table 2**) in 300 µl of buffer 1. Then, 15µl of pre-washed Protein A agarose (GeneScript, Piscataway, NJ, USA) were added, and samples were incubated for an additional 4 h at 4^0^C with rotation. Beads were then washed four times with buffer 1 before elution of protein complexes using the SDS-PAGE Sample buffer (Thermo Fisher Scientific, Waltham, MA, USA). After that, samples were processed as described above.

### 2.9 Detection of catalytically active proteasome subunits

Visualization of catalytically active proteasome subunits was performed using the Me_4_BodipyFL-Ahx_3_Leu_3_VS (UbiQbio, Amsterdam, The Netherlands) activity-based probe. The probe was used in accordance with the previously published protocol (40). Briefly, cellular homogenates were incubated with the 1 µM of the probe for 2 h at 37^0^C. After that, fluorescent proteasome subunits were detected following the electrophoresis in 12% Tris-Glicine polyacrylamide SDS gel using the ChemiDoc XRS+ imaging system (Bio-Rad, Hercules, CA, USA). The BodipyFL fluorescence was detected at an excitation wavelength of 480 nm and an emission wavelength of 530 nm. To verify equal protein loading, the gel was incubated with the ROTI^®^Blue quick protein stain (Carl Roth, Karlsruhe, Germany).

### 2.10 Fluorescent and confocal microscopy

All cell lines were routinely monitored using the Evos FL (Thermo Fisher Scientific, Waltham, MA, USA) fluorescent microscope. In addition, the fluorescence of SW620, SW620B8-mCherry, TZM-blB8-mCherry, SW620B8-mCherryB5-GFP and TZM-blB8-mCherryB10-CFP cells was analyzed 72 h after the incubation with 1000 U/mL of recombinant human IFN-γ (R&D systems, Minneapolis, MN, USA), and 500 U/mL of recombinant human TNF (R&D systems, Minneapolis, MN, USA). To prepare SW620B8-mCherryB5-GFP and TZM-blB8-mCherryB10-CFP cells for confocal microscopy, cells were grown in the Clipmax culture flasks (TPP, Trasadingen, Switzerland). 24 h after seeding, recombinant human IFN-γ (1000 U/mL) (R&D systems, Minneapolis, MN, USA) and recombinant human TNF (500 U/mL) (R&D systems, Minneapolis, MN, USA) were added to the cell culture media. The cells remained in the incubator for an additional 72 h. Then, cells were washed twice with PBS and fixed in a 4% PFA (BosterBio, Pleasanton, CA, USA) for 15 min. To visualize cellular nuclei, the SW620B8-mCherryB5-GFP cells were washed with PBS and incubated with the NucBlue Fixed Cell ReadyProbe (Thermo Fisher Scientific, Waltham, MA, USA) for 15 min. Finally, cells were covered with SlowFade™ Glass Antifade Mountant (Thermo Fisher Scientific, Waltham, MA, USA) and cover slips (Thermo Fisher Scientific, Waltham, MA, USA). The Leica DMI 6000 B microscope with the Leica TCS SP5 laser scanning unit (Leica, Mannheim, Germany) was used to analyze the prepared slides. Photoconversion of PS-CFP2 was induced by intense 400 nm light irradiation. Fluorescence of the photoconverted protein was observed at 511 nm after excitation with 488 nm light using the Nikon Ti2 Eclipse microscope (Nikon, Nishi-Ōi, Shinagawa, Tokyo, Japan). The Coloc 2 plugin FIJI (ImageJ) software^4^ was used for pixel co-localization analysis. Mean intensity values of pixels (0–255 for 8-bit images) were measured within the nuclei of cells after photoconversion. For the microscopy of living cells, the cells were grown in 35 mm cell culture dishes and stimulated with pro-inflammatory cytokines as described above. Cells were examined using multimodal nonlinear imaging (see below).

### 2.11 Tumor inoculation and *in vivo* imaging

A total of 3x10^5^ SW620 or SW620B8-mCherryB5-GFP cells in 100 μL PBS were injected subcutaneously into the right and the left flank of 6-week-old NSG-SGM3 mice. Tumor growth was monitored for 28 days using optical tomography LumoTrace^®^ FLUO bioimaging system (Abisense, Sirius Federal Territory, Russia) on a weekly basis. Imaging parameters were as follows: mCherry fluorescence — exposure 5000 ms, binning 1 × 1, LED 590 x 2, filter 650-40. The isolated tumors were imaged using the following parameters: mCherry fluorescence — exposure 1000 ms, binning 1 × 1, LED 590 x 2, filter 650-40; GFP fluorescence - exposure 1000 ms, binning 1 × 1, LED 470 x 4, filter 550-40. The Icy software (Version 2.5.2.0) (Institut Pasteur and France-BioImaging, Paris, France), was used to quantify fluorescent signals.

### 2.12 Multimodal nonlinear imaging

Two-photon excitation fluorescence (2PEF) imaging of fluorescent tags EGFP and mCherry was performed on a nonlinear microscope MPM-200 (Thorlabs, Newton, NJ, USA). The second harmonics microscopy and multiphoton excited endogenous chromophores imaging were also provided. A detailed description of the multimodal imaging system has been published previously (41, 42). Two ultrafast lasers independently pumped the microscope. The first was a titanium:sapphire (Ti:S) laser Mira HP (Coherent, Santa Clara, CA, USA) pumped by a 20 W continuous wave (CW) frequency-doubled ytterbium-doped fiber amplifier at 532 nm (Precilasers, Jiading District, Shanghai, China) producing transform-limited pulses at repetition rate of 76 MHz with a duration of 90 fs at a central wavelength of 880 nm and an energy up to 43 nJ. The second source was a diode-pumped solid-state ytterbium-ion (Yb) ultrafast laser TEMA (Avesta Project Ltd., Moscow, Russia), providing 110 fs pulses at a central wavelength of 1045 nm with energy of about 45 nJ. The central wavelengths of the lasers were set to provide the most selective excitation of EGFP and mCherry tags, in accordance with their quantitative two-photon absorption spectra (43, 44).

Laser beams were combined into a single spatial mode on a dichroic mirror DMSP1000 (Thorlabs, Newton, NJ, USA). Tight beam focusing was achieved with a water-dipping infrared (IR) microscope objective XLPLN25XWMP2 x25 1.05NA (Olympus, Shinjuku, Tokyo, Japan). In order to manage the objective back pupil filling and light transmission to the sample, each individual laser path was equipped with telescopes and mechanical shutters. The fluorescence emission was separated from the pump radiation by a dichroic mirror FF775-Di01 (Semrock, Rochester, NY, USA) and a short-pass edge filter FF01-745/SP-25 (Semrock, Rochester, NY, USA). A nonlinear signal registration unit consisted of a “blue channel” selecting band of 430–490 nm (FELH0400 and FESH0500, Thorlabs, Newton, NJ, USA) for NAD(P)H emission and second harmonics detection; a “green channel” of 500–550 nm (FELH0500 and FESH0550, Thorlabs, Newton, NJ, USA) for EGFP emission; and a “red channel” of 595–665 nm (MF630-69, Thorlabs, Newton, NJ, USA) for mCherry emission. Low noise, high sensitive photomultiplier tubes (PMTs) H10721–210 and H7422PA-40 (Hamamatsu photonics, Shizuoka, Japan) were used.

In experiments with cell cultures and tissue samples, the average pump power was approximately 20 mW and 100 mW for Ti:S and Yb lasers, respectively, measured at the entrance to the microscope using a thermal power meter S401C (Thorlabs, Newton, NJ, USA). Transmission of the optical system, including the microscope and the objective, at pump wavelengths of 880 nm and 1045 nm, was about 75% and 55%, respectively. The transverse and axial resolution of the 2PEF microscopy were about 0.4 μm nm and 1.4 μm, respectively, at the both wavelengths. Multiphoton images were acquired with a dwell time of 1.4 µs, a frame averaging about 5 and a resolution of 1024x1024 pixels. Quantitative comparison of cellular and tissue EGFP and mCherry fluorescence was performed using the Origin2018 software (OriginLab Corporation, Northampton, MA, USA).

### 2.13 Flow cytometry

Twenty-four hours after seeding, the cell lines were stimulated with 1000 U/mL of recombinant human IFN-γ (R&D systems, Minneapolis, MN, USA) and 500 U/mL of recombinant human TNF (R&D systems, Minneapolis, MN, USA). After a 72 h-long incubation, cells were washed twice with PBS and detached from the surface using a trypsin-EDTA solution (PanEko, Moscow, Russia). Detached cells were mixed with fresh culture media, centrifuged for 5 min at 250 x g, and cellular pellets were resuspended in 500 µL of PBS. The LSRFortessa flow cytometer (BD Biosciences, Franklin Lakes, NJ, USA) was used to characterize the cellular fluorescence. All tests were performed in at least triplicates. The obtained data were analyzed using the FlowJo software version 10.0.7 (FlowJo LLC, Ashland, OR, USA; RRID: SCR_008520), and GraphPad Prism software version 8.4.3. (GraphPad Software, San Diego, CA, USA; RRID: SCR_002798).

### 2.14 Statistics

The statistical analysis of the results was performed using the t-test and the GraphPad Prism software version 8.4.3 (“GraphPad Software”, USA), * p < 0.05; ** - p < 0.01; *** - p < 0.001; **** -p < 0.0001.

## 3 Results

### 3.1 Increased GFP and CFP fluorescence is observed following genome editing of SW620B8-mCherry and TZM-blB8-mCherry cell lines

Genome editing was performed to enhance the functionality of previously generated reporter cell lines SW620B8-mCherry and TZM-blB8-mCherry. The Cas9D10A was chosen to increase the specificity of genome editing and minimize potential off-target effects. To enable visualization of cPs in SW620B8-mCherry cells, a sequence encoding enhanced green fluorescent protein (EGFP) was inserted in frame at the 3’ end of the *PSMB5* gene, which encodes the constitutive β5 proteasome subunit (**Figure 1A**). To distinguish iPs from intPs in TZM-blB8-mCherry cells, a sequence encoding photoswitchable fluorescent protein PS-CFP2 was introduced in frame at the 3’ end of the *PSMB10* gene, encoding the β2i immunoproteasome subunit (**Figure 1A**). Both cell lines were transfected with pDG461-based vectors and donor plasmids. After three consecutive rounds of sorting and expansion of the transfected SW620B8-mCherry cells, we obtained a population in which 99% of cells exhibited a 10-fold increase in mean EGFP fluorescence (p<0.001) (**Figure 1B, C**). This cell line was annotated as SW620B8-mCherryB5-GFP. Six rounds of propagation together with repeated IFN-γ/TNF stimulation and sorting were used to obtain a population of transfected TZM-blB8-mCherry cells in which 91% of cells demonstrated 3-fold increase (p<0.0001, t-test) in PS-CFP2 fluorescence after treatment with cytokines (**Figures 1B, C**). The novel cell line was referred to as TZM-blB8-mCherryB10-CFP. Both edited cell lines showed more than 20-fold (SW620B8-mCherryB5-GFP) and almost 40-fold (TZM-blB8-mCherryB10-CFP) increase (p<0.0001, t-test) in mean mCherry fluorescence following incubation with IFN-γ and TNF (**Figures 1B, C**). This indicates preservation of the mechanisms regulating β5i-mCherry expression in the modified cells. Notably, analysis of EGFP fluorescence in SW620B8-mCherryB5-GFP cells revealed more than 20% decrease (p<0.0001, t-test) in fluorescence after stimulation with IFN-γ and TNF (**Figure 1C**), reflecting a possible replacement of cPs with iPs in cytokine-treated cells.

### 3.2 Newly generated cell lines contain full-length and correctly positioned genomic inserts

To assure that the observed increases in intracellular EGFP and PS-CFP2 fluorescence were due to the expression of chimeric genes, genomic DNA was isolated from control and modified cells. PCR with primer sets targeting the 5’ and 3’ regions of the inserts, along with unmodified genomic DNA, confirmed successful genome modification in SW620B8-mCherryB5-GFP and TZM-blB8-mCherryB10-CFP cell lines (**Supplementary Figure 2**). No specific bands were detected in samples from control SW620 and TZM-bl cells. Sequencing of entire chimeric transcripts confirmed the expected nucleotide composition and demonstrated the absence of mutations.

### 3.3 The expression of *PSMB5-GFP* and *PSMB10-CFP* chimeras is regulated by endogenous regulatory mechanisms in genetically modified cells

**Figure 2:**
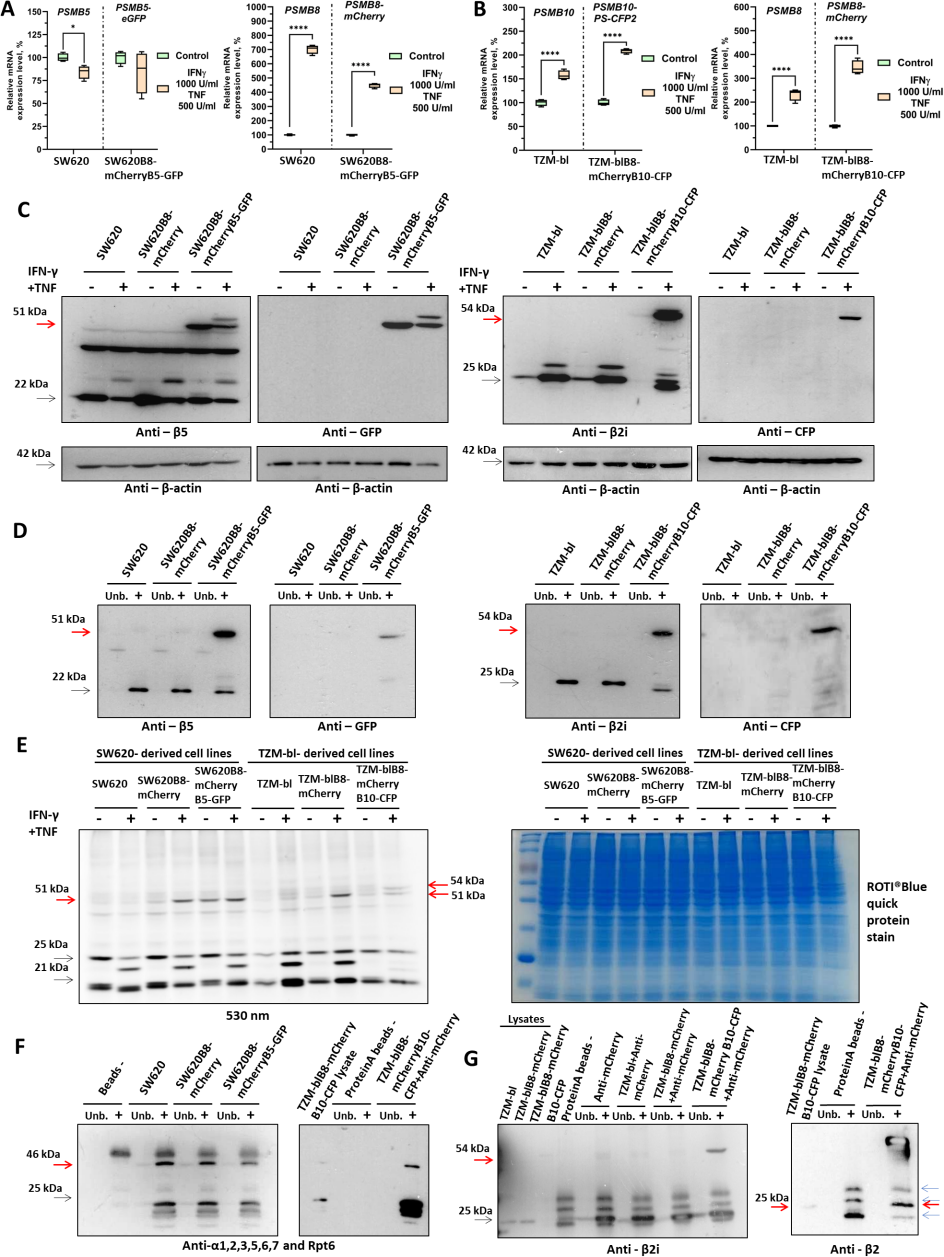
Validation of SW620B8-mCherryB5-GFP and TZM-blB8-mCherryB10-CFP cell lines. **(A)** Relative expression levels of the *PSMB5, PSMB8* genes in SW620 and the *PSMB5-EGFP*, *PSMB8-mCherry* chimeras in SW620B8-mCherryB5-GFP cells, respectively. Gene expression was assessed in control and cytokine-treated (IFN-γ (1000 U/mL), TNF (500 U/mL)) cells. **(B)** Relative expression levels of the *PSMB10*, *PSMB8*, genes in TZM-bl cells and the *PSMB10-PS-CFP2*, *PSMB8-mCherry* genes in TZM-blB8-mCherryB10-CFP cells. mRNA samples were obtained from control and IFN-γ/TNF-treated cells. Primers to endogenous *PSMB5*, *PSMB8* and *PSMB10* genes were used for PCR with mRNA from initial cell lines, while primers targeting chimeras were utilized to study gene expression in modified cells (**Supplementary Table 1**^38^). *p < 0.05; ****p < 0.0001; t-test. **(C)** (Left) Western blotting of lysates obtained from SW620, SW620B8-mCherry and SW620B8-mCherryB5-GFP, and the same cells incubated with 1000 U/mL IFN-γ and 500 U/mL TNF for 72 h. (Right) Western blotting of lysates obtained from unstimulated TZM-bl, TZM-blB8-mCherry and TZM-blB8-mCherryB10-CFP, and the same cells treated with 1000 U/mL IFN-γ and 500 U/mL TNF for 72 h. To ensure an equal protein load, the membranes were stripped and then incubated with anti-β-actin antibodies. For PS-CFP detection, anti-GFP antibodies (Cell Signaling, Boston, MA, USA) were used. **(D)** (Left) Western blotting of proteasomes immunoprecipitated via the α4 subunit from homogenates of SW620 and derived cell lines using a Proteasome purification kit (Enzo, Farmingdale, NY, USA). (Right) Western blotting of proteasomes immunoprecipitated via α4 subunits from homogenates of TZM-bl and derived cell lines treated with IFN-γ/TNF for 72 h. The Proteasome purification kit (Enzo) was used to isolate proteasomes. The PS-CFP2 was revealed using anti-GFP (Cell Signaling) antibodies. The unbound fractions are marked as Unb. **(E)** Visualization of catalytically active proteasome subunits in cellular lysates. Homogenates of unstimulated and cytokine-treated (1000 U/mL IFN-γ and 500 U/mL TNF for 72 h) control and modified cells were incubated for 2 h at 37 °C with the Me_4_BodipyFL-Ahx_3_Leu_3_VS proteasome activity-based probe. The catalytic proteasome subunits were identified in 12% Tris-Glycine polyacrylamide gel following the excitation at a wavelength of 480 nm and an emission wavelength of 530 nm (left panel). The right panel shows the same gel stained with ROTI^®^Blue quick protein stain (Carl Roth, Karlsruhe, Germany). **(F)** (Left) Western blotting of proteasomes immunoprecipitated via the α4 subunit from homogenates of SW620 and derived cell lines using a Proteasome purification kit (Enzo). Anti-mouse heavy chain HRP conjugates were used as secondary antibodies (ABclonal, Woburn, MA, USA). (Right) Western blotting of proteasomes immunoprecipitated from homogenates of IFN-γ/TNF-treated TZM-blB8-mCherryB10-CFP cells using antibodies to mCherry (Abcam, Cambridge, UK). Protein A agarose beads were used for the immunoprecipitation. **(G)** (Left) Western blotting of cellular homogenates and proteasomes immunoprecipitated using Protein A resin and anti-mCherry antibodies (Abcam) from homogenates of TZM-bl and derived cell lines, treated with IFN-γ/TNF for 72 h. Anti-rabbit light chain HRP conjugates (Cell Signaling) were used to detect primary antibody binding. (Right) Western blotting of TZM-bl-derived cell lines homogenates and proteasomes immunoprecipitated using Protein A resin and anti-mCherry antibodies (Abcam) from lysates of cytokine-treated TZM-blB8-mCherryB10-CFP cells. *Anti-rabbit heavy chain HRP conjugates (GeneTex, Irvine, CA, USA) were used as secondary antibodies. Specific bands are shown by red arrows and unspecific bands are shown by blue arrows.

Flow cytometry of IFN-γ and TNF-stimulated cells revealed decreased EGFP fluorescence and increased PS-CFP2 fluorescence in SW620B8-mCherryB5-GFP and TZM-blB8-mCherryB10-CFP cells, respectively (**Figures 1B, C**). Because pro-inflammatory cytokines upregulate immunoproteasome subunit expression (12), while suppressing synthesis of constitutive proteasome subunits (45), observed changes in cellular fluorescence may be attributed to this effect. To verify that expression of *PSMB5-EGFP* and *PSMB10-PS-CFP2* was altered accordingly, the qPCR was performed using specific primer sets. It has been shown that following proinflammatory cytokine stimulation, the expression of endogenous PSMB5 was slightly decreased in SW620 cells (p<0.05 t-test) and the expression of chimeric PSMB5-EGFP showed a similar, but not statistically significant trend in SW620B8-mCherryB5-GFP cells. In contrast, the expression of *PSMB10* and *PSMB10-PS-CFP2* was significantly increased in TZM-bl (p<0.0001, t-test) and TZM-blB8-mCherryB10-CFP cells (p<0.0001, t-test, respectively) after treatment with IFN-γ and TNF (**Figures 2A, B**). At the same time, the magnitude of changes in the expression of chimeric genes did not match that of wild-type genes. This effect may be attributed to unintended epigenetic alterations arising at the edited locus following CRISPR/Cas9-mediated DNA cleavage and repair (46). However, since modifications were introduced at the 3’ ends of the genes, consequences for transcriptional regulation are presumably less pronounced. Overall, the observed co-directional changes in gene expression in both control and modified cells indicate that endogenous regulatory mechanisms controlling *PSMB5-EGFP* and *PSMB10-PS-CFP2* expression are preserved.

### 3.4 Chimeric β5-GFP and β2i-CFP proteins are incorporated into proteasomes and are capable of performing proteolysis in SW620B8-mCherryB5-GFP and TZM-blB8-mCherryB10-CFP cells

We next assessed the presence of the chimeric proteins in lysates of control and modified cells by Western blotting. Using antibody pairs recognizing proteasome subunits and fluorescent proteins, we demonstrated presence of chimeric proteins with expected molecular weights (~51 kDa for β5-EGFP and ~54 kDa for β2i-PS-CFP2) in edited cell lines, but not in control cells (**Figure 2C**). Notably, endogenous proteasome subunits were also present in homogenates of modified cells, indicating the presence of heterozygous populations within the obtained cell lines. Moreover, consistent with the data described in sections 3.1 and 3.3, we observed upregulation of β2i-PS-CFP2 and downregulation of β5-EGFP expression following treatment of cells with pro-inflammatory cytokines. In addition to the mature chimeric proteins, Western blotting revealed less intense bands with slightly greater molecular weights than 51 and 54 kDa, which likely correspond to uncleaved precursor forms of the proteasome subunits. Importantly, no free PS-CFP2 or EGFP were detected in the cellular lysates.

To verify the incorporation of chimeric subunits into proteasomes, immunoprecipitation of proteasomes via the α4 subunit was performed (**Figure 2D**). Proteasomes isolated from SW620B8-mCherryB5-GFP and TZM-blB8-mCherryB10-CFP cells contained chimeric β5-EGFP and β2i-PS-CFP2 subunits, confirming their integration into assembled proteasomes. Finally, chimeric subunits efficiently bound the Me_4_BodipyFL-Ahx_3_Leu_3_VS activity-based probe, indicating their catalytically active state and retained proteolytic function (**Figure 2E**).

### 3.5 Fluorescent proteasomes associate with 19S proteasome activators

The degradation of ubiquitinated proteins is primarily performed by the 26S proteasome, which consists of the 20S core particle associated with one or two 19S activators. To determine whether proteasomes in modified cells retain their ability to bind to 19S activators, we immunoprecipitated proteasomes from SW620, SW620B8-mCherry and SW620B8-mCherryB5-GFP cells using agarose beads coated with anti-α4 subunit antibodies (**Figure 2F** left). Western blotting revealed that the immunoprecipitated samples contained comparable amounts of proteasome α subunits, as well as the Rpt6 subunit of the 19S activator, indicating equal association of proteasomes with 19S activators in the tested cell lines. To address the association of modified proteasomes with 19S activators in TZM-blB8-mCherryB10-CFP cells, the immunoprecipitation of proteasomes from IFN-γ/TNF-stimulated cells was performed using anti-mCherry antibodies (**Figure 2F** right). The presence of Rpt6 subunit in the obtained fraction demonstrated that at least some of the immunoprecipitated proteasomes interact with the 19S activator.

### 3.6 Chimeric subunits β5i-mCherry and β2i-PS-CFP2 integrate into the same proteasomes in TZM-blB8-mCherryB10-CFP cells

To determine whether both fluorescent subunits β5i-mCherry and β2i-PS-CFP2 are incorporated into the same proteasomes in TZM-blB8-mCherryB10-CFP cells, we performed immunoprecipitation of proteasomes from IFN-γ/TNF-stimulated TZM-bl, TZM-blB8-mCherry and TZM-blB8-mCherryB10-CFP cells using anti-mCherry antibodies. Western blot analysis with antibodies to the β2i subunit revealed the presence of the chimeric β2i-PS-CFP2 subunit in samples from TZM-blB8-mCherryB10-CFP cells (**Figure 2G** left). This confirms that at least a portion of proteasomes in TZM-blB8-mCherryB10-CFP cells contain both chimeric subunits simultaneously. The presence of intPs among immunoprecipitated proteasomes was confirmed by Western blotting using antibodies to the constitutive β2 subunit (**Figure 2G** right). Thus, it was demonstrated that in cytokine-treated TZM-blB8-mCherryB10-CFP cells, β5i-mCherry-containing proteasomes are both iPs and intPs.

### 3.7 Immune and intermediate proteasomes accumulate in the nuclei of TZM-blB8mCherryB10-CFP cells following stimulation with pro-inflammatory cytokines

**Figure 3:**
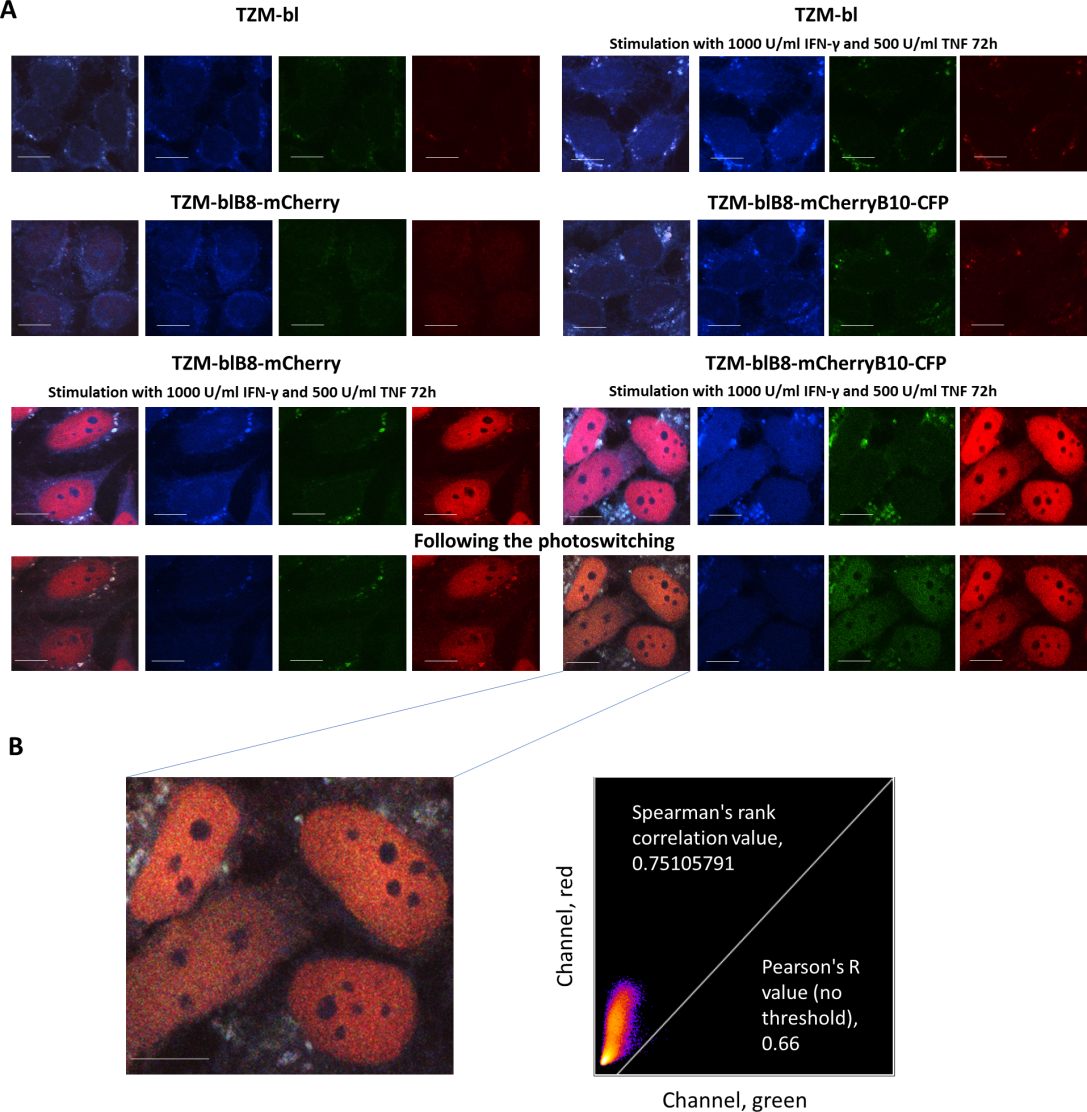
Immune and intermediate proteasomes accumulate in the nuclei of TZM-blB8mCherryB10-CFP cells following stimulation with pro-inflammatory cytokines. **(A)** Confocal microscopy of unstimulated TZM-bl, TZM-blB8-mCherry, TZM-blB8-mCherryB10-CFP cells, and the same cells treated with pro-inflammatory cytokines. Twenty four hours after seeding, 1000 U/mL of recombinant human IFN-γ and 500 U/mL of recombinant human TNF were added to the culture medium and the cells were incubated for an additional 72 h. After that, the cells were fixed in a 4% PFA solution. The photoconversion of PS-CFP2 was induced by intense 400 nm light irradiation. The fluorescence of the photoconverted protein was detected at 511 nm following excitation with a 488 nm light. Images are given in order from left to right: merged image, PS-CFP2 (blue channel), EGFP (green channel), mCherry (red channel). The fluorescence of PS-CFP2 can be seen in blue before photoconversion and in green after photoconversion. **(B)** (Left) The TZM-blB8-mCherryB10-CFP cells after photoconversion at a higher magnification. (Right) The Coloc 2 plugin FIJI (ImageJ) software^4^ was used to perform pixel co-localization analysis, measuring the mean intensity value of pixels (0–255 for 8-bit images) within cell nuclei following photoconversion. The scale bar is 10 µm.

As the β2i subunit is predominantly incorporated into iPs rather than into intPs (5), photoswitching of β2i-PS-CFP2 allows the localization of iPs in modified cells following exposure to pro-inflammatory cytokines. Confocal microscopy of control and IFN-γ/TNF-stimulated TZM-bl, TZM-blB8-mCherry, and TZM-blB8mCherryB10-CFP cells (**Figure 3A**) revealed an increase in nuclear mCherry fluorescence in TZM-blB8-mCherry and TZM-blB8mCherryB10-CFP cells upon cytokine treatment. Moreover, an elevated PS-CFP2 fluorescence was detected in the nuclei of TZM-blB8mCherryB10-CFP cells (**Figure 3A**). Notably, nucleoli lacked detectable fluorescence, indicating the absence of proteasomes in these structures. To specifically visualize β2i-containing proteasomes, irreversible photoconversion was induced by using intense 400 nm light irradiation. As expected, no photoconversion occurred in control TZM-bl-mCherry cells. In contrast, robust photoswitching was observed predominantly in the nuclei of TZM-blB8mCherryB10-CFP cells. Notably, more “red” signals were detected rather than an overlay of red and green fluorescence, resulting in “yellow”. This suggests that intPs are more abundant than iPs in the nuclei of these cells. The co-localization of β5i-mCherry (red) and photoswitched β2i-PS-CFP2 (green) signals was analyzed using the Coloc 2 plugin of ImageJ software (**Figure 3B**). A high, but not complete, degree of red and green pixel co-localization was revealed (Spearman’s rank correlation value, 0.75105791 and Pearson’s R value (no threshold), 0.66). Consistent with this, distinct “green-only” spots were observed, which likely correspond to β2i-PS-CFP2-containing proteasomes, lacking β5i-mCherry. Together, the predominance of red signals and the absence of 100% co-localization of mCherry and PS-CFP2 indicate that cells accumulate both iPs and intPs in their nuclei. It is also worth noting that TZM-bl and related cell lines frequently contained blue and, to a lesser extent, green fluorescent aggregates. However, these structures did not undergo photoswitching.

### 3.8 Intracellular distribution of constitutive and non-constitutive proteasomes in SW620B8-mCherryB5-GFP cells

**Figure 4:**
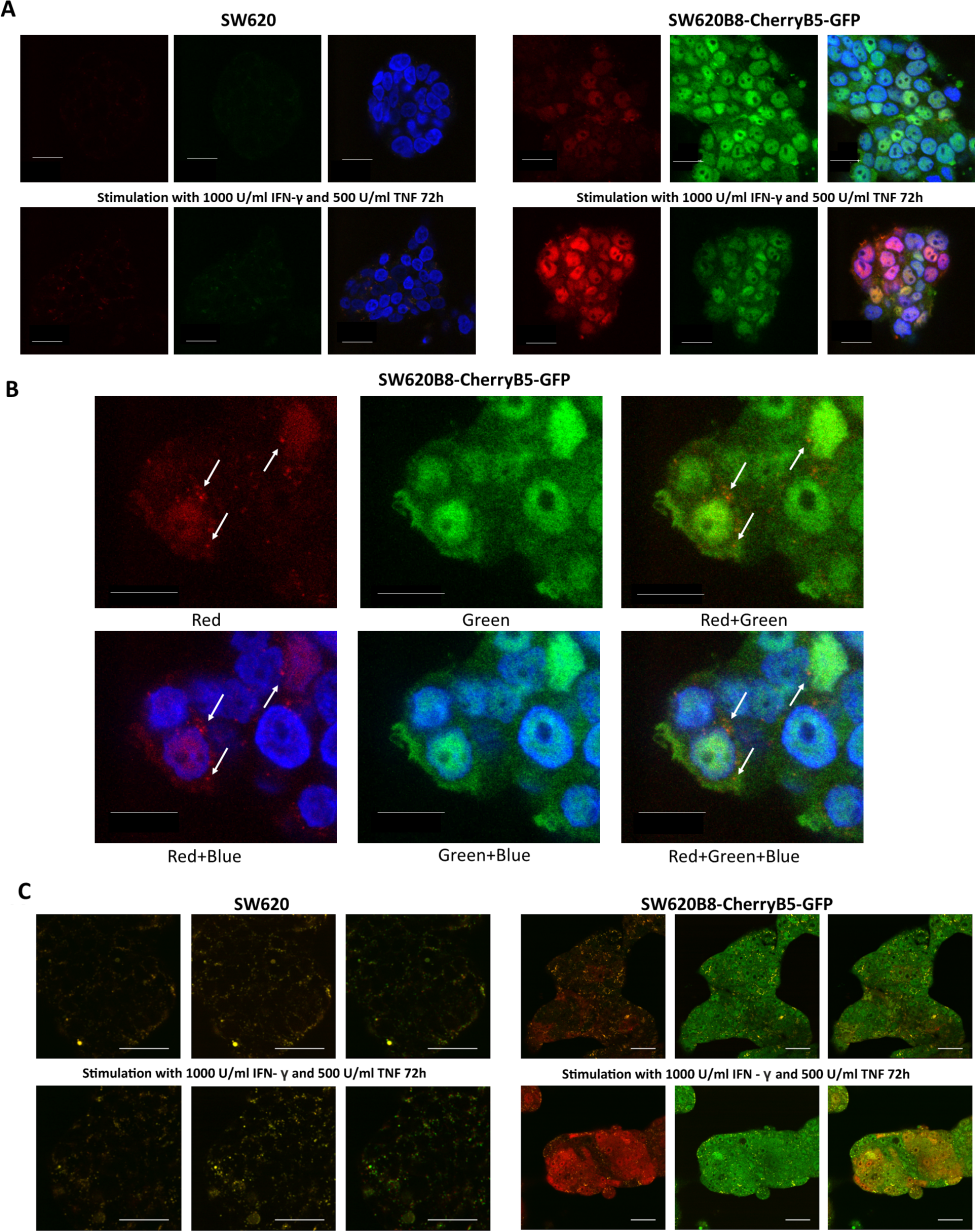
Non-constitutive proteasomes form aggregates within the SW620B8-mCherryB5-GFP cells. **(A)** Confocal microscopy of unstimulated or IFN-γ/TNF-treated SW620 and SW620B8-mCherryB5-GFP cells. Pro-inflammatory cytokines (1000 U/mL of IFN-γ and 500 U/mL of TNF) were added to the culture media and the cells were left in the incubator for 72 h. After that, cells were fixed in a 4% PFA solution. To visualize cellular nuclei, cells were incubated with the NucBlue Fixed Cell ReadyProbe (Thermo Fisher Scientific, Waltham, MA, USA) (blue fluorescence). The images are given in order from left to right: mCherry (red channel), EGFP (green channel), merged image. Scale bar – 25 µm. **(B)** Magnification of unstimulated SW620B8-mCherryB5-GFP cells. Different combinations of fluorescence channels are shown. Non-constitutive proteasome aggregates are indicated by white arrows. The mCherry fluorescence is shown in red, the EGFP – in green. Merging of particular channels is indicated by “+”. Cellular nuclei are shown in blue. Scale bar – 10 µm. **(C)** Multiphoton microscopy of unstimulated or IFN-γ/TNF-treated live SW620 (left) and SW620B8-mCherryB5-GFP (right) cells. The nonlinear microscopy setup was optimized for the most distinct imaging of EGFP and mCherry, which was achieved by alternately two-photon excitation of the sample with pulses at wavelengths of 880 nm and 1045 nm, with emission detection in orthogonal spectral channels at 500–550 nm and 590–665 nm. The images are presented in order from left to right: mCherry (red channel), EGFP (green channel), and merged image. The SW620 and SW620B8-mCherryB5-GFP cells were cultured in DMEM lacking phenol red. Scale bar – 25 µm.

To characterize the intracellular localization of constitutive and non-constitutive proteasomes, we performed confocal microscopy of paraformaldehyde-fixed SW620, SW620B8-mCherry and SW620B8-mCherryB5-GFP cells, either unstimulated or treated with pro-inflammatory cytokines. As expected, only SW620B8-mCherryB5-GFP cells displayed intense EGFP fluorescence (**Figure 4A**). Stimulation with IFN-γ and TNF resulted in marked increase in mCherry fluorescence in SW620B8-mCherryB5-GFP cells. At the same time, the mCherry fluorescence level varied among individual cells. In most cytokine-stimulated cells, enhanced mCherry emission was accompanied by a modest reduction in EGFP signal, indicating a reorganization of the proteasome pool towards an increase in the quantity of non-constitutive proteasomes and a decrease in constitutive proteasome expression (**Figure 4A**). Notably, no EGFP or mCherry fluorescence was detected in the control SW620 cell line. In SW620B8-mCherryB5-GFP cells, both constitutive and non-constitutive proteasomes were predominantly localized within the nucleus. Treatment with IFN-γ and TNF further stimulated nuclear accumulation of non-constitutive proteasomes. A more detailed analysis of intracellular proteasome distribution revealed that, in unstimulated SW620B8-mCherryB5-GFP cells, non-constitutive proteasomes frequently accumulated in small optically dense foci or aggregate-like structures (**Figure 4B**). This observation is consistent with our previous findings, where non-constitutive proteasome-enriched aggregates were detected in SW620B8-mCherry cells (34). However, analysis of the SW620B8-mCherryB5-GFP cell line revealed that constitutive proteasomes were largely absent from these structures, as the foci exhibited only weak co-localization of EGFP and mCherry signals (**Figure 4B**). This observation suggests that distinct proteasome forms may follow different intracellular localization patterns.

To investigate proteasome localization in living cells, we employed the multiphoton microscopy. Compared with conventional confocal microscopy, multiphoton imaging offers several advantages, including compatibility with live tissue observation, improved imaging depth, and reduced photodamage (47). The nonlinear microscopy setup was optimized to distinctly visualize EGFP and mCherry. This was achieved by alternating two-photon excitation of the sample with pulses at 880 nm and 1045 nm and detecting emissions in orthogonal spectral channels at 500–550 nm and 590–665 nm, respectively. Such an arrangement effectively eliminated cross-channel interference, achieving a suppression ratio of approximately four orders of magnitude.

The SW620 and SW620B8-mCherryB5-GFP cells were cultured in phenol red-free DMEM and either stimulated with IFN-γ and TNF or left untreated. As expected, SW620B8-mCherryB5-GFP exhibited significantly increased EGFP fluorescence compared to control cells. In addition, exposure of the modified cells to pro-inflammatory cytokines led to a significant increase in the mCherry signal (**Figure 4C**). Notably, mCherry induction within the SW620B8-mCherryB5-GFP population, was heterogeneous, with some cells displaying considerably higher expression than others. Despite this variability, the overall fluorescence patterns and intracellular proteasome distribution were consistent with those observed using confocal microscopy (**Figure 4A**).

### 3.9 Non-constitutive proteasomes are upregulated within tumor xenografts in response to IFN-γ

**Figure 5:**
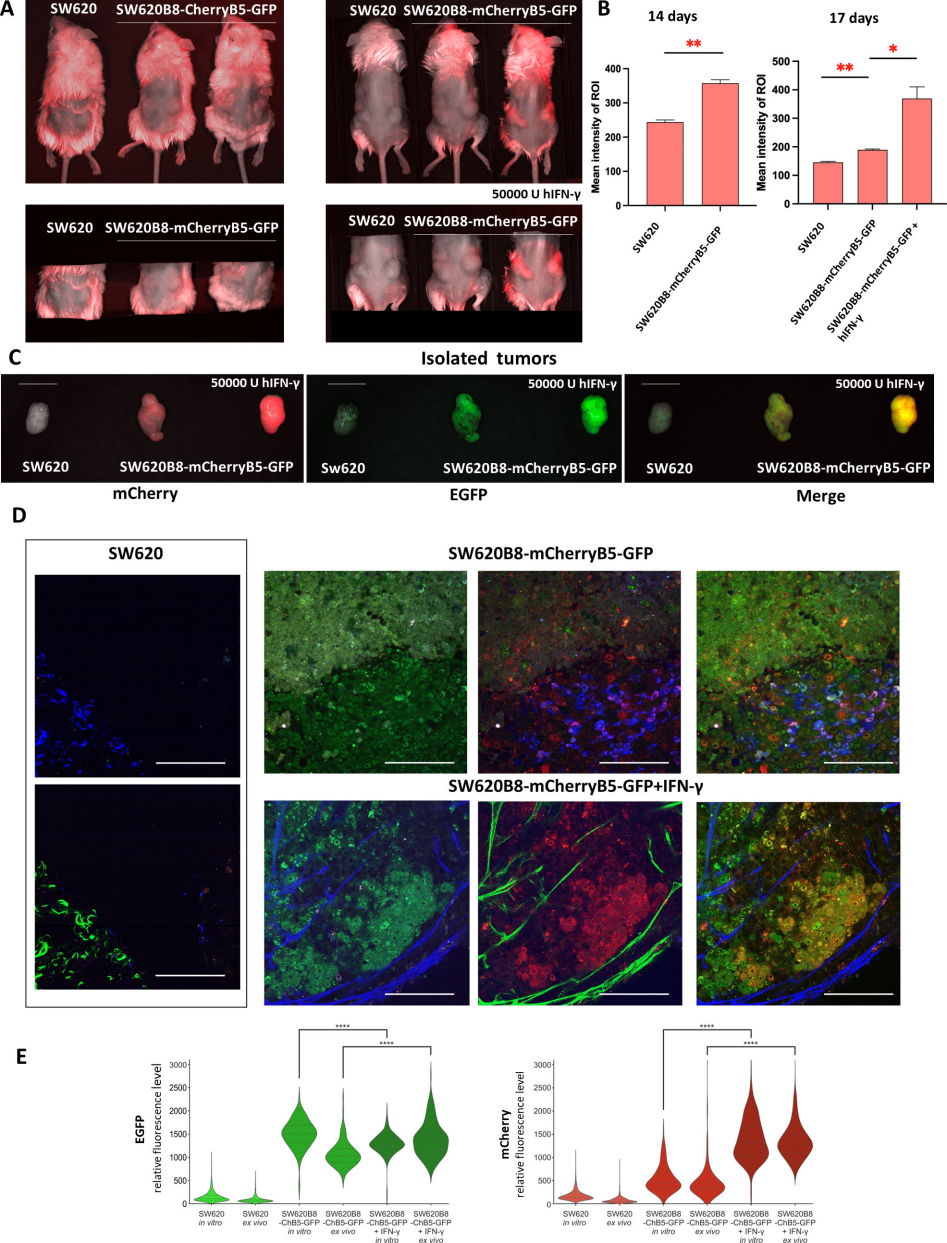
Non-constitutive proteasomes are upregulated in tumor xenografts in response to IFN-γ. **(A)***In vivo* imaging of NSG-SGM3 mice subcutaneously injected with 300.000 of control SW620 or SW620B8-mCherryB5-GFP cells. (Left) Mice 14 days after the inoculation of cancer cells. (Right) The same mice, 17 days after the injection of tumor cells; one of the animals was stimulated with 50.000 U of human IFN-γ for 72 h **(B)** Comparative analysis of mCherry fluorescence intensity in mice injected with SW620 and SW620B8-mCherryB5-GFP cells 14 days following injection of tumor cells (left). Comparative analysis of mCherry fluorescence in mice injected with SW620 and SW620B8-mCherryB5-GFP cells 17 days after tumor cell inoculation. Where indicated, animals were stimulated with 50.000 U of human IFN-γ for 72 h (right). *p < 0.05; **p < 0.01; t-test. **(C)** The imaging of representative tumors obtained from animals injected with SW620 or SW620B8-mCherryB5-GFP cells. Where indicated, animals were administrated with 50.000 U of human IFN-γ. The mCherry, EGFP or merge fluorescence is shown. **(D)** Multiphoton microscopy images of tumors shown in **(C)**. Scale bar – 100 µm. **(E)** Comparison of cellular (**Figure 4C**) and tissue (**Figure 5D**) EGFP and mCherry fluorescence determined using the Origin 2018 software. ****p < 0.0001, t-test.

To investigate the distribution of constitutive and non-constitutive proteasomes *in vivo* we subcutaneously injected 300.000 of control SW620 or SW620B8-mCherryB5-GFP cells into immunodeficient NSG-SGM3 mice. Animals were monitored twice per week, and visible tumors developed 14 days after inoculation. Using an *in vivo* imaging system, we observed that tumors derived from SW620B8-mCherryB5-GFP cells exhibited 1.5-fold higher (p <0.01, t-test) mCherry fluorescence compared to tumors formed by SW620 cells (**Figures 5A, B**; **Supplementary Figure 4**). EGFP fluorescence could not be detected due to the sensitivity limitations of the *in vivo* imaging system (data not shown). To assess whether non-constitutive proteasome expression in tumor cells can be induced by pro-inflammatory cytokines, SW620B8-mCherryB5-GFP tumor-bearing NSG-SGM3 mice received an intraperitoneal injection of 50.000 U hIFN-γ. After 72 h, tumors in IFN-γ-treated mice showed a 2-fold increase (p<0.05, t-test) in mCherry fluorescence compared to SW620B8-mCherryB5-GFP-derived carcinomas in unstimulated mice (**Figures 5A, B**; **Supplementary Figure 5**). Following imaging, animals were euthanized and perfused with PBS. Tumors were isolated and again analyzed using a fluorescent imager. As expected, SW620B8-mCherryB5-GFP-derived tumors demonstrated from 3- to 8-fold increase in mCherry fluorescence and a 2.5- to 5-fold increase in EGFP fluorescence compared to control SW620-derived tumors (**Figure 5C**; **Supplementary Figure 6**). Tumor samples were further analyzed using multiphoton microscopy.

**Figure 6:**
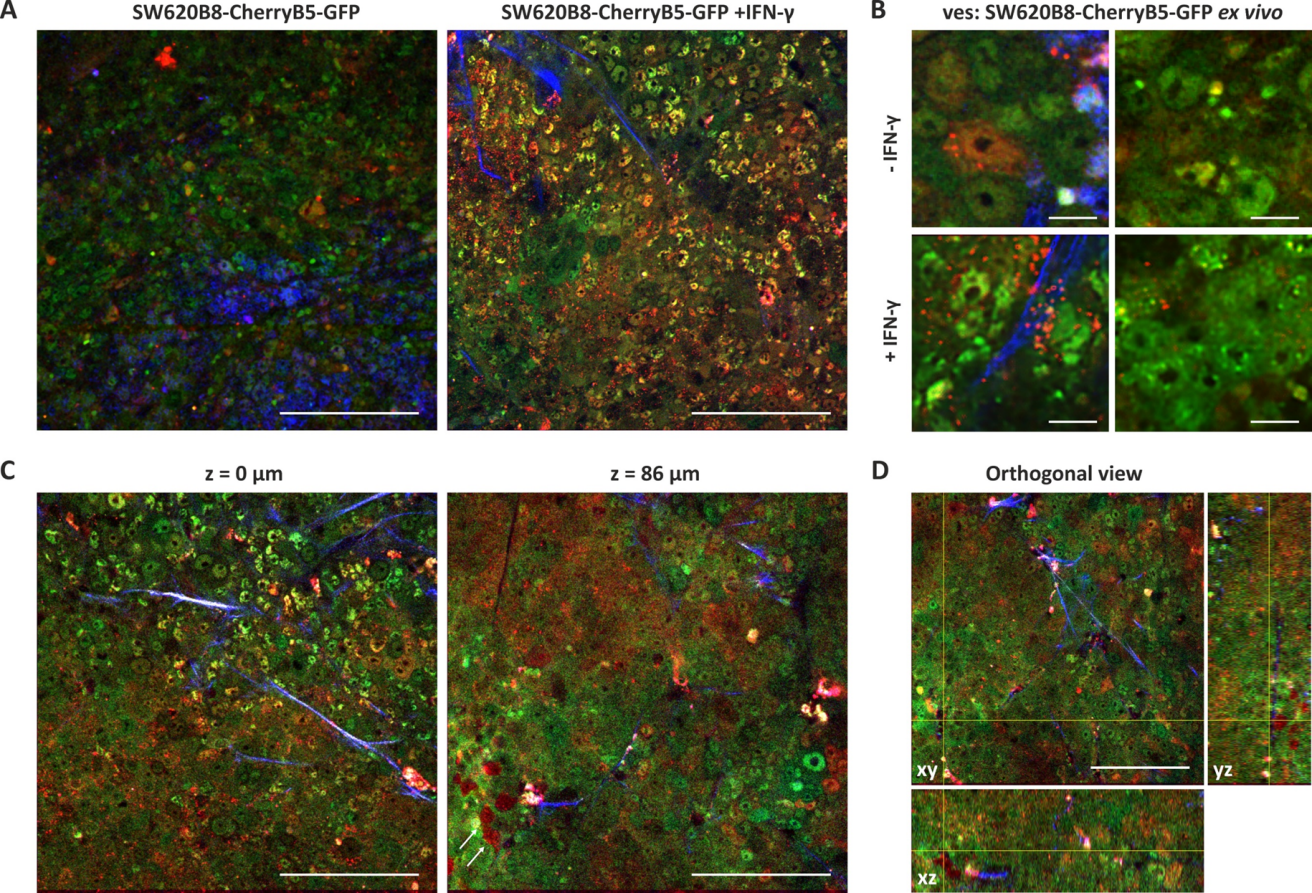
Non-constitutive proteasomes are likely to be secreted from the tumor xenografts in response to IFN-γ. **(A)** Multiphoton microscopy of tumors derived from SW620B8-mCherryB5-GFP cells. Left panel - untreated animal. Right panel – mice injected with 50.000 U of human IFN-γ. Scale bar – 100 µm. **(B)** Magnification of tumor tissue images from SW620B8-mCherryB5-GFP-injected control and IFN-γ-treated mice. Spherical objects with bright red fluorescence can be seen both inside and outside cells. Scale bar – 10 µm. **(C)** Multiphoton microscopy of a tumor derived from an animal injected with SW620B8-mCherryB5-GFP and treated with human IFN-γ at the surface (left panel) and at the depth of 86 µm (right panel). Arrows indicate cells with spherical structures, containing non-constitutive proteasomes. Scale bar – 100 µm. **(D)** Orthogonal view of a slice produced by two-color multiphoton microscopy. Scale bar – 50 µm.

Nonlinear fluorescent imaging was enhanced by second harmonic generation microscopy, which is readily recognized by repetitive patterns appearing in different spectral channels depending on the pump central wavelength. The second harmonic clearly revealed collagen fibers visible as blue or green structures in connective tissue, the tumor’s superficial capsule, and the angiogenic vascular network. As expected, tumors derived from SW620 cells exhibited no specific fluorescence signal and minimal autofluorescence (**Figure 5D**). In contrast, tumors obtained from SW620B8-mCherryB5-GFP-bearing mice displayed intense EGFP and mCherry emission, exceeding the signal from control tissues by 12- and 6-fold (p < 0.0001, t-test), respectively. This increase was comparable to the fluorescence elevation observed in cell culture, where EGFP and mCherry intensities rose more than 8-fold and 7-fold (p < 0.0001, t-test), respectively (**Figure 5E**). Within the tumors, EGFP signal was evenly distributed among cells in the proliferating regions of the tissue, producing a symmetrical probability distribution. In contrast, mCherry fluorescence exhibited a long-tailed distribution, revealing a subset of bright cells. Notably, tumors derived from SW620B8-mCherryB5-GFP cells also showed bright signal of NAD(P)H cofactors in the blue channel (430–490 nm), which was detected by the emerging three-photon excitation band at a wavelength of 1045 nm during NAD(P)+ reduction (48) (**Figure 5D**, **6A**).

Following exposure of tumor-bearing mice to hINF-γ, the fluorescence pattern of tumors changed significantly. The intensity of mCherry fluorescence increased by approximately 3-fold (p < 0.0001, t-test), indicating robust induction of non-constitutive proteasome synthesis. A moderate but significant 1.3-fold increase in the EGFP signal was also detected (p < 0.0001, t-test) (**Figures 5D, E**). This result contrasts with the dynamics observed in cell cultures by flow cytometry and two-photon excitation microscopy, where EGFP fluorescence decreased by 1.3-fold (p < 0.0001, t-test) and 1.2-fold (p < 0.0001, test), respectively (**Figures 4**, **5E**). Notably, the induction of non-constitutive proteasomes within tumors was heterogenous: while some cells demonstrated bright fluorescence, others showed relatively weak fluorescence (**Figures 5D**, **6A**). Additionally, in contrast to carcinomas from untreated mice, the NAD(P)H signal was not detected within the tumors (**Figures 5D**, **6A**). As NAD(P)+/NAD(P)H serves as a central metabolic hub controlling cellular redox homeostasis, the NAD(P)H provides the reducing power for antioxidant defense (49). Thus, observed differences in the NAD(P)H signal suggest significant alterations in the redox balance of tumor cells following the IFN-γ treatment.

Notably, we detected numerous “vesicles” of different sizes (0.7 - 3 μm) with bright mCherry fluorescence within the nuclei, cytoplasm and extracellular space in SW620B8-mCherryB5-GFP-derived tumors, but not in tumors originating from SW620 cells. These structures were present at a density of approximately 10^3^ mm^−2^ (**Figures 5D**, **6A–D**). Following IFN-γ treatment, their abundance increased dramatically, reaching 1–5 ×10^4^ mm^−2^. Although some green and yellow “vesicles” were detected, the majority displayed exclusively mCherry fluorescence. The strongest release of red “vesicles” was observed in tumor regions, where individual cell morphology became indistinguishable and the fluorescent signal appeared “homogeneous”. In the EGFP channel, while cells retained green fluorescence, similar inclusions were found nearly 100 times less frequently, indicating preferential enrichment of non-constitutive proteasomes in the “vesicles” generated by tumor cells. Analysis of deeper tissue layers revealed a greater proportion of “vesicles” located inside the cells (**Figures 6C, D**; **Supplementary Movie 1**), suggesting that a few of these structures, initially identified as extracellular, may have been released from damaged cells during sample preparation. Nevertheless, “vesicles” clearly localized between cells were still observed.

## 4 Discussion

Since the discovery of distinct proteasome forms (19, 20, 50), considerable effort has been devoted to elucidating their specific roles. Functional differences between cPs and iPs have been underscored by their predominant expression in specific cellular populations, their regulation by modulatory molecules, and ability to generate altering peptide sets (5, 19, 20, 51). Compared to cPs, iPs and intPs exhibit increased chymotrypsin-like activity, expand the diversity of produced peptides, are highly enriched in immune cells, and are strongly upregulated in many other cell types in response to pro-inflammatory cytokines (8, 17, 26, 52, 53). Hence, their role in MHC class I-dependent antigen presentation and immune reactions is well established. In addition, iPs have been shown to regulate T lymphocyte expansion (54) and cytokine production (27, 55). At the same time, these proteasomes participate in several key signaling pathways beyond classical immunological cascades, such as visual signal transmission (56), maintenance of stem cell pluripotency (57), and muscle differentiation (58). Furthermore, iPs modulate the transcription of more than 8000 different genes in dendritic cells (59). Although some studies do not clearly distinguish between iPs and intPs, the available evidence highlights functional differences between constitutive and non-constitutive proteasomes. Consistently, distinct proteasome forms demonstrate varying efficiencies in degrading oxidized and damaged proteins, illustrating their specific contributions to cellular stress responses (25). This is further supported by findings that certain cell types can simultaneously express cPs, iPs and intPs independent of cytokine stimulation (5, 60).

Interestingly, various cancer cells also express different proteasome subtypes. At the same time, upregulation of iPs in the tumor can have paradoxical consequences. On the one hand, elevated iP expression may enhance the presentation of neoantigens and thereby facilitate immune recognition of tumor cells (30). On the other hand, increased iP activity may support cancer progression by helping tumor cells cope with proteotoxic stress, accelerating the degradation of tumor suppressor proteins, and sustaining a pro-inflammatory microenvironment (55, 61). Consistent with this, mice lacking β5i exhibit resistance to chronic inflammation and the development of colitis-associated cancer (55).

Proteasomes are well-established targets in cancer therapy. Currently, three clinically approved inhibitors: bortezomib (Velcade), carfilzomib (Kyprolis) and ixazomib (Ninlaro), directed towards all major proteasome forms are used for the treatment of hematological malignancies (62). However, proteasome-inhibitor-based therapies face several important challenges, including the development of drug resistance (63), limited efficacy against solid tumors (64) and strong side effects (65). Systemic side effects could potentially be reduced by using compounds that selectively target specific proteasome forms. In particular, inhibitors directed toward iP subunits may offer therapeutic advantages in certain cancers. For example, the β5i-selective inhibitor ONX-0914 reduces tumor burden in a colitis-associated cancer model (55). In line with the regulatory effects of iPs on the immune response, this inhibitor and its analogue have also shown efficacy in several autoimmune disease models (27, 31, 66–68). Phase 1b/2 clinical trial evaluating the ONX-0914 analogue KZR-616 was recently completed (https://www.clinicaltrials.gov/study/NCT03393013). Collectively, these results underscore the importance of elucidating the distinct biological functions of proteasome subtypes. Addressing this challenge requires the development of advanced experimental tools and approaches capable of elucidating the roles of specific proteasome forms and assessing the impact of pharmacological compounds on the cellular proteasome pool.

Here, we developed and validated two novel cell lines that allow visualization of different proteasome forms within a single cell. We confirmed that both cell lines contain full-length, correctly integrated genomic inserts and express proteasome subunits fused to fluorescent proteins. Importantly, the chimeric proteins are efficiently incorporated into proteasomes and can perform proteolysis. Preservation of endogenous regulatory mechanisms together with robust induction of the chimeric β5i and β2i subunits upon IFN-γ treatment indicates that SW620B8-mCherryB5-GFP and TZM-blB8-mCherryB10-CFP cells are suitable for monitoring the effects of various compounds, including proteasome stimulators and inhibitors, on immune subunit expression using rapid, high-throughput screening assays. This capability may facilitate the identification of novel modulators of non-constitutive proteasome synthesis, which could in turn enhance anti-tumor immune responses and suppress tumor progression (30). Moreover, the absence of “free” fluorescent proteins and the minimal abundance of precursor chimeric subunits in cell lysates indicate that the fluorescent subunits are integrated into assembled proteasomes, allowing for precise localization of specific proteasome subtypes within cells, or if they are secreted – outside cells. Thus, the colorectal adenocarcinoma cell line SW620B8-mCherryB5-GFP allowed us, for the first time, to simultaneously visualize the localization of constitutive and non-constitutive proteasomes within a cancer cell. We found that, in addition to their diffuse distribution throughout the nucleus and cytoplasm, non-constitutive proteasomes form dense perinuclear areas that are devoid of cPs (**Figure 4B**). Similar structures were previously reported (34), yet the presence or absence of constitutive proteasomes within these structures have not been clarified. The nature of the non-constitutive proteasome-enriched areas remains unresolved. Also, it is not clear whether they correspond to stress granules (69), proteasome storage granules, stress-associated proteasome foci (70), or specialized sites of proteasome assembly or degradation. In addition, the localization of non-constitutive proteasomes to specific cell compartments may reflect their functional specialization, potentially influencing the repertoire of antigens generated for MHC class I presentation or indicating their involvement in endoplasmic reticulum (ER)-associated degradation (71). We cannot entirely exclude the possibility that formation of these structures is influenced by the fluorescent protein tags, but no comparable structures were observed for the β5-EGFP chimera. Future studies using organelle-specific markers and antibodies to characteristic proteins will be crucial for determining the exact nature of these non-constitutive proteasome-enriched structures. In addition to relatively small foci enriched in non-constitutive proteasomes, we observed an increased number of larger spherical cellular inclusions with bright mCherry fluorescence in SW620B8-mCherryB5-GFP-derrived tumors in NSG-SGM3 mice (**Figures 5D**, **6**). Both the abundance of non-constitutive proteasomes and the amount of inclusions containing them were dramatically increased following IFN-γ stimulation. This finding not only confirms the suitability of our model for visualizing the *in vivo* effects of various stimuli on the proteasome pool, but also underscores the predominant incorporation of non-constitutive proteasomes into these “aggregates”, as most inclusions exhibited exclusively mCherry fluorescence. Strikingly, we also detected numerous red “aggregates” in the extracellular space, suggesting their possible secretion in response to pro-inflammatory cytokine exposure (**Figures 5D**, **6**). These structures may represent extracellular vesicles (EVs), and, given their size, they may be either microvesicles or apoptotic bodies shed from cells producing non-constitutive proteasomes. Previously, several studies demonstrated that SW620 cells actively secrete EVs (72–75), and they are enriched in proteins associated with cancer progression (72). Subsequent studies identified MTOR, PRKCA, MACC1, MARCKS, FGFR4 and other oncogenic proteins within these vesicles, as well as proteins implicated in the formation of pre-metastatic niche. Functionally, EVs from SW620 cells were shown to affect recipient fibroblasts: EV-treated fibroblasts exhibited enhanced invasion through Matrigel (74), and underwent metabolic reprogramming from a quiescent state to cancer-associated fibroblast phenotype (73). This activation was associated with the upregulation of pro-invasive regulators of membrane protrusion and matrix remodeling proteins, consistent with a role for EVs in shaping a pro-tumorigenic microenvironment (73). Moreover, SW620-derived EVs have been reported to influence immune cells, increasing CD14 expression and inducing the synthesis of IL-6, CXCL10, IL-23, and IL-10 in M0 macrophages, as well as the production of IL-23 in M2 polarized macrophages (76). Therefore, EVs derived from SW620 cells appear to contribute not only to cancer progression and metastasis, but also to the modulation of the tumor microenvironment. Interestingly, accumulating data indicate increased levels of extracellular proteasomes in the serum of patients with various pathologies, including cancer (77). Multiple studies have demonstrated that proteasomes can be efficiently secreted via EVs (13, 21, 78–81). Recent findings further show that EV-mediated delivery of 20S proteasomes can promote the degradation of toxic proteins in recipient cells (82). Moreover, we have recently identified proteasomes within retroviral particles (83). Together, these observations suggest that EV-associated proteasomes may play important roles in intercellular communication, maintenance of tissue homeostasis, protection or, conversely, may contribute to pathogen dissemination and cancer progression. These functions may include stimulation of antigen presentation, degradation of toxic proteins or protein aggregates, hydrolysis of cellular proteins such as restriction factors, processing of viral or host proteins within virions or vesicles, generation of biologically-active peptides, activation of signaling pathways and many other processes. Nevertheless, the precise biological purpose of proteasome secretion and particularly the reason why the “vesicles” observed in our experiments contain mostly non-constitutive proteasomes remains unclear. It is important to note that cells with high levels of non-constitutive proteasome expression still retain substantial amounts of “green” constitutive proteasomes, however, the latter generally do not appear to aggregate or become secreted. This observation raises the intriguing possibility that excessive accumulation of non-constitutive proteasomes may be toxic to cells, at least within the tissue context. This interpretation is supported by recent findings, highlighting that the build-up of non-constitutive proteasomes induces toxicity and apoptosis in inflamed neurons (84). Although we cannot fully exclude that some vesicles originate from damaged or dying cells, our results nevertheless point to potentially specific and diverse roles of constitutive and non-constitutive proteasomes in cancer cells. Additional experiments will be required to elucidate the mechanisms underlying these phenomena.

Compared to cPs and iPs, the biological role of intPs remains largely enigmatic. These proteasomes contain β1i and/or β5i along with constitutive subunits (5) (**Table 1**). IntPs have been detected in multiple organs, including the liver, colon, and bone marrow, as well as in specific immune cell types, such as granulocytes, monocytes and dendritic cells (4, 5, 51). Notably, intPs were shown to generate unique peptide repertoires from cancer-associated antigens (85). Moreover, they reside in pancreatic β cells, where their synthesis is upregulated by low concentrations of IL-1β (86). Despite these intriguing observations, studies focusing specifically on intPs remain scarce.

To establish an appropriate model for studying intPs, we generated a new cell line, TZM-blB8-mCherryB10-CFP. These cells were obtained using the previously reported TZM-blB8-mCherry cell line (34) as a backbone, and co-express the immune subunits β2i and β5i tagged with different fluorescent proteins. It should be noted that β2i tagging carries a potential risk, given the reported significance of the C-terminus of β2 subunit in proteasome assembly (87). However, our immunoprecipitation experiments (**Figures 2D, G**) and analysis using the activity-based probe (**Figure 2E**) indicate that active proteasomes containing PS-CFP2-tagged β2i subunits can indeed be formed. Since most intPs reportedly lack the β2i subunit (5), this cell line enables visual discrimination between iPs and intPs. Moreover, fusing β2i to the photoswitchable protein PS-CFP2, allows real-time tracking of iPs in living cells using live-cell imaging. Indeed, fusion of a photoconvertible protein to a proteasome subunit has previously been used to monitor the shuttling of proteasomes between the nucleus and the cytoplasm under conditions of amino acid deprivation (88). In our study, we analyzed TZM-blB8-mCherryB10-CFP cells after IFN-γ treatment and subsequent photoconversion. We found that fluorescent proteasomes were concentrated within the nuclei, and most of these proteasomes showed “red” fluorescence, indicating the incorporation of the β5i-mCherry subunit. Only a minority appeared “yellow”, containing both β5i-mCherry and β2i-PS-CFP2 subunits. This suggests that the intPs are more abundant than iPs within the modified cells. At the same time, we cannot exclude the possibility that the reduced “yellow” signal arises from proteasomes that incorporate unmodified β2i subunits, since cells still possess low level of endogenous subunit expression (**Figures 2C, D**). Interestingly, in addition to “red” and “yellow” signals, a small number of “green” spots were also detected. Considering that proteasome assembly generally occurs outside the nucleus (89–91) and that our data suggest minimal amounts of β2i-PS-CFP2 precursor protein (**Figure 2C**), these “green” structures may represent a small population of intPs that contain β2i-PS-CFP2, but lack β5i-mCherry. Indeed, atypical intPs comprising β1i/β2i/β5 subunit set were previously reported in IFN-γ-stimulated cells lacking β5i (92). Thus, our findings support the possibility that limited amounts of such alternative intPs can assemble even in the presence of β5i. However, further studies will be required to confirm this observation.

At the same time, our study has certain limitations. First, fluorescent protein tagging may influence proteasome activity or its interaction with activators. To address this, we demonstrated that the chimeric subunits bind the activity-based probe, indicating that they are incorporated into assembled proteasomes and remain catalytically active (**Figure 2E**). Moreover, we revealed that immunoprecipitated proteasomes from the modified cells were associated with 19S activators (**Figure 2F**). Our previous data also show only marginal differences in total proteasome activity between SW620 and SW620B8-mCherry, as well as between TZM-bl and TZM-blB8-mCherry cells (34). Collectively, these observations suggest that tagging of selected proteasome β subunits with fluorescent proteins does not impair their integration into proteasomes and has little, if any, effect on the association of 20S proteasomes with 19S activators. However, a direct side-by-side comparison of the modified proteasomes with purified endogenous cPs, iPs, and intPs was not performed. Therefore, we cannot exclude the possibility that fusion to fluorescent proteins may affect post-translational modifications of proteasome subunits or alter interactions with other activators or associated proteins. Another limitation of our study is related to the use of the CRISPR/Cas9 genome editing technique. Although we used mutant Cas9D10A and performed rational design of gRNAs, we cannot entirely exclude the possibility of off-target genome editing effects, which could somehow affect the expression of cellular genes. Moreover, since each cell line possesses unique biological characteristics, results obtained in one cell line may not fully translate to others. Accordingly, caution should be exercised when generalizing findings, especially in the context of xenograft models. It is also important to note that our *in vivo* experiments were performed in immunocompromised mice. Due to their severely reduced immune cell populations, these animals do not allow robust evaluation of the interactions between tumor cells and immune system components, including potential roles of distinct proteasome forms in these processes. Future studies employing humanized mice or engineered murine cell lines compatible with immunocompetent hosts may help overcome this limitation.

Altogether, the SW620B8-mCherryB5-GFP and TZM-blB8-mCherryB10-CFP cell lines possess unique properties that make them powerful tools for monitoring proteasome pool diversity both *in vitro* and *in vivo*, under basal conditions and following treatment with pharmacological agents, including proteasome stimulators or inhibitors. As a result, these cell lines may facilitate the identification of novel anti-cancer compounds targeting specific proteasome forms. Furthermore, the established cell-based platform allows real-time visualization of proteasome dynamics in living cells, enabling detailed analysis of the distinct contributions of cPs, iPs, and intPs to cellular homeostasis and intercellular communication. Finally, the cell lines have the potential to provide important insights into the mechanisms underlying cancer progression, cellular adaptation to different conditions, and modulation of immune-related processes.

## Data Availability

The raw data supporting the conclusions of this article will be made available by the authors, without undue reservation.

## Footnotes

1
https://crispr.bme.gatech.edu/

2
http://www.rgenome.net/cas-offinder/

3
https://eu.idtdna.com

4
https://imagej.nih.gov/ij/

## References

[1] Ciechanover A Schwartz AL . The ubiquitin-proteasome pathway: the complexity and myriad functions of proteins death. Proc Natl Acad Sci USA. (1998) 95:2727–30. doi: 10.1073/pnas.95.6.2727, PMID: 9501156 PMC34259

[2] Groll M Ditzel L Löwe J Stock D Bochtler M Bartunik HD . Structure of 20S proteasome from yeast at 2.4 A resolution. Nature. (1997) 386:463–71. doi: 10.1038/386463a0, PMID: 9087403

[3] Fabre B Lambour T Garrigues L Ducoux-Petit M Amalric F Monsarrat B . Label-free quantitative proteomics reveals the dynamics of proteasome complexes composition and stoichiometry in a wide range of human cell lines. J Proteome Res. (2014) 13(6):3027–37. doi: 10.1021/pr500193k, PMID: 24804812

[4] Gohlke S Kloss A Tsokos M Textoris-Taube K Keller C Kloetzel PM . Adult human liver contains intermediate-type proteasomes with different enzymatic properties. Ann Hepatol. (2014) 13:429–38. doi: 10.1016/S1665-2681(19)30850-6, PMID: 24927614

[5] Guillaume B Chapiro J Stroobant V Colau D Van Holle B Parvizi G . Two abundant proteasome subtypes that uniquely process some antigens presented by HLA class I molecules. Proc Natl Acad Sci USA. (2010) 107:18599–604. doi: 10.1073/pnas.1009778107, PMID: 20937868 PMC2972972

[6] Kinoshita M Hamakubo T Fukui I Murachi T Toyohara H . Significant amount of multicatalytic proteinase identified on membrane from human erythrocyte. J Biochem. (1990) 107:440–4., PMID: 2187858 10.1093/oxfordjournals.jbchem.a123064

[7] Klare N Seeger M Janek K Jungblut PR Dahlmann B . Intermediate-type 20 S proteasomes in HeLa cells: “asymmetric subunit composition Diversity adaptation. J Mol Biol. (2007) 373:1–10. doi: 10.1016/j.jmb.2007.07.038, PMID: 17804016

[8] Mishto M Liepe J Textoris-Taube K Keller C Henklein P Weberruss M . Proteasome isoforms exhibit only quantitative differences in cleavage and epitope generation. Eur J Immunol. (2014) 44:3508–21. doi: 10.1002/eji.201444902, PMID: 25231383

[9] Pelletier S Schuurman KG Berkers CR Ovaa H Heck AJ Raijmakers R . Quantifying cross-tissue diversity in proteasome complexes by mass spectrometry. Mol Biosyst. (2010) 6:1450–3. doi: 10.1039/c004989a, PMID: 20498902

[10] Vigneron N Van den Eynde BJ . Proteasome subtypes and regulators in the processing of antigenic peptides presented by class I molecules of the major histocompatibility complex. Biomolecules. (2014) 4(4):994–1025. doi: 10.3390/biom4040994, PMID: 25412285 PMC4279167

[11] Fabre B Lambour T Garrigues L Amalric F Vigneron N Menneteau T . Deciphering preferential interactions within supramolecular protein complexes: the proteasome case. Mol Syst Biol. (2015) 11:771. doi: 10.15252/msb.20145497, PMID: 25561571 PMC4332148

[12] Aki M Shimbara N Takashina M Akiyama K Kagawa S Tamura T . Interferon-gamma induces different subunit organizations and functional diversity of proteasomes. J Biochem. (1994) 115:257–69. doi: 10.1093/oxfordjournals.jbchem.a124327, PMID: 8206875

[13] Bochmann I Ebstein F Lehmann A Wohlschlaeger J Sixt SU Kloetzel PM . T lymphocytes export proteasomes by way of microparticles: a possible mechanism for generation of extracellular proteasomes. J Cell Mol Med. (2014) 18:59–68. doi: 10.1111/jcmm.12160, PMID: 24304442 PMC3916118

[14] Pickering AM Linder RA Zhang H Forman HJ Davies KJ . Nrf2-dependent induction of proteasome and Pa28alphabeta regulator are required for adaptation to oxidative stress. J Biol Chem. (2012) 287:10021–31. doi: 10.1074/jbc.M111.277145, PMID: 22308036 PMC3323025

[15] Rouette A Trofimov A Haberl D Boucher G Lavallee VP D’Angelo G . Expression of immunoproteasome genes is regulated by cell-intrinsic and -extrinsic factors in human cancers. Sci Rep. (2016) 6:34019. doi: 10.1038/srep34019, PMID: 27659694 PMC5034284

[16] St-Pierre C Morgand E Benhammadi M Rouette A Hardy MP Gaboury L . Immunoproteasomes Control the Homeostasis of Medullary Thymic Epithelial Cells by Alleviating Proteotoxic Stress. Cell Rep. (2017) 21(9):2558–70. doi: 10.1016/j.celrep.2017.10.121, PMID: 29186691

[17] Zittlau KI Zachor-Movshovitz D Leushkin Y Schimmel Brener R Morgenstern D Ben-Nissan G . Tracking proteasome degradation: A cross-organ analysis via intact degradomics mass spectrometry. Proc Natl Acad Sci U S A. (2025) 122:e2419607122. doi: 10.1073/pnas.2419607122, PMID: 39964708 PMC11874349

[18] Visekruna A Joeris T Schmidt N Lawrenz M Ritz JP Buhr HJ . Comparative expression analysis and characterization of 20S proteasomes in human intestinal tissues: The proteasome pattern as diagnostic tool for IBD patients. Inflammation Bowel Dis. (2009) 15:526–33. doi: 10.1002/ibd.20805, PMID: 19067411

[19] Driscoll J Brown M Finley D Monaco JJ . MHC-linked LMP gene products specifically alter peptidase activities of the proteasome. Nature. (1993) 365:262–4. doi: 10.1038/365262a0, PMID: 8371781

[20] Akiyama K Yokota K Kagawa S Shimbara N Tamura T Akioka H . cDNA cloning and interferon-γ down-regulation of proteasomal subunits X and Y. Science. (1994) 265:1231–4. doi: 10.1126/science.8066462, PMID: 8066462

[21] Morozov A Karpov V . Biological consequences of structural and functional proteasome diversity. Heliyon. (2018) 4:e00894. doi: 10.1016/j.heliyon.2018.e00894, PMID: 30417153 PMC6218844

[22] Morozov AV Karpov VL . Proteasomes and several aspects of their heterogeneity relevant to cancer. Front Oncol. (2019) 9:761. doi: 10.3389/fonc.2019.00761, PMID: 31456945 PMC6700291

[23] Seifert U Bialy LP Ebstein F Bech-Otschir D Voigt A Schröter F . Immunoproteasomes preserve protein homeostasis upon interferon-induced oxidative stress. Cell. (2010) 142:613–24. doi: 10.1016/j.cell.2010.07.036, PMID: 20723761

[24] Winter MB La Greca F Arastu-Kapur S Caiazza F Cimermancic P Buchholz TJ . Immunoproteasome functions explained by divergence in cleavage specificity and regulation. Elife. (2017) 6:e27364. doi: 10.7554/eLife.27364, PMID: 29182146 PMC5705213

[25] Abi Habib J De Plaen E Stroobant V Zivkovic D Bousquet MP Guillaume B . Efficiency of the four proteasome subtypes to degrade ubiquitinated or oxidized proteins. Sci Rep. (2020) 10:15765. doi: 10.1038/s41598-020-71550-5, PMID: 32978409 PMC7519072

[26] Abi Habib J Lesenfants J Vigneron N Van den Eynde BJ . Functional differences between proteasome subtypes. Cells. (2022) 11:421. doi: 10.3390/cells11030421, PMID: 35159231 PMC8834425

[27] Muchamuel T Basler M Aujay MA Suzuki E Kalim KW Lauer C . A selective inhibitor of the immunoproteasome subunit LMP7 blocks cytokine production and attenuates progression of experimental arthritis. Nat Med. (2009) 15:781–7. doi: 10.1038/nm.1978, PMID: 19525961

[28] Dantuma NP Bott LC . The ubiquitin-proteasome system in neurodegenerative diseases: precipitating factor, yet part of the solution. Front Mol Neurosci. (2014) 7:70. doi: 10.3389/fnmol.2014.00070, PMID: 25132814 PMC4117186

[29] Zhou X Xu R Wu Y Zhou L Xiang T . The role of proteasomes in tumorigenesis. Genes Dis. (2023) 11:101070. doi: 10.1016/j.gendis.2023.06.037, PMID: 38523673 PMC10958230

[30] Rana PS Ignatz-Hoover JJ Guo C Mosley AL Malek E Federov Y . Immunoproteasome activation expands the MHC class I immunopeptidome, unmasks neoantigens, and enhances T-cell anti-myeloma activity. Mol Cancer Ther. (2024) 23:1743–60. doi: 10.1158/1535-7163.MCT-23-0931, PMID: 39210605 PMC11612626

[31] Muchamuel T Fan RA Anderl JL Bomba DJ Johnson HWB Lowe E . Zetomipzomib (KZR-616) attenuates lupus in mice via modulation of innate and adaptive immune responses. Front Immunol. (2023) 14:1043680. doi: 10.3389/fimmu.2023.1043680, PMID: 36969170 PMC10036830

[32] Reits EA Benham AM Plougastel B Neefjes J Trowsdale J . Dynamics of proteasome distribution in living cells. EMBO J. (1997) 16:6087–94. doi: 10.1093/emboj/16.20.6087, PMID: 9321388 PMC1326292

[33] Schipper-Krom S Sanz AS van Bodegraven EJ Speijer D Florea BI Ovaa H . Visualizing proteasome activity and intracellular localization using fluorescent proteins and activity-based probes. Front Mol Biosci. (2019) 6:56. doi: 10.3389/fmolb.2019.00056, PMID: 31482094 PMC6710370

[34] Burov A Funikov S Vagapova E Dalina A Rezvykh A Shyrokova E . A cell-based platform for the investigation of immunoproteasome subunit β5i expression and biology of β5i-containing proteasomes. Cells. (2021) 10:3049. doi: 10.3390/cells10113049, PMID: 34831272 PMC8616536

[35] Burov A Rezvykh A Vedernikova V Belogurov A Prassolov V Spirin P . Caffeine modulates immunoproteasome activity and content in colorectal adenocarcinoma cells. Biochimie. (2025) 235:1–13. doi: 10.1016/j.biochi.2025.05.004, PMID: 40349826

[36] Wunderlich M Chou FS Link KA Mizukawa B Perry RL Carroll M . AML xenograft efficiency is significantly improved in NOD/SCID-IL2RG mice constitutively expressing human SCF, GM-CSF and IL-3. Leukemia. (2010) 24:1785–8. doi: 10.1038/leu.2010, PMID: 20686503 PMC5439963

[37] Billerbeck E Barry WT Mu K Dorner M Rice CM Ploss A . Development of human CD4+FoxP3+ regulatory T cells in human stem cell factor-, granulocyte-macrophage colony-stimulating factor-, and interleukin-3-expressing NOD-SCID IL2Rγ(null) humanized mice. Blood. (2011) 117:3076–86. doi: 10.1182/blood-2010-08-301507, PMID: 21252091 PMC3062310

[38] Morozov AV Burov AV Astakhova TM Spasskaya DS Margulis BA Karpov VL . Dynamics of the functional activity and expression of proteasome subunits during cellular adaptation to heat shock. Mol Biol. (2019) 53:571–9. doi: 10.1134/S0026893319040071, PMID: 31397437

[39] Funikov SY Spasskaya DS Burov AV Teterina EV Ustyugov AA Karpov VL . Structures of the Mouse Central Nervous System Contain Different Quantities of Proteasome Gene Transcripts. Mol Biol (Mosk). (2021) 55(1):54–63. doi: 10.31857/S002689842006004X, PMID: 33566025

[40] De Jong A Schuurman KG Rodenko B Ovaa H Berkers CR . Fluorescence-based proteasome activity profiling. Methods Mol Biol. (2012) 803:183–204. doi: 10.1007/978-1-61779-364-6_13, PMID: 22065226

[41] Lanin A Chebotarev A Kelmanson I Pochechuev M Fetisova E Bilan D . Single-beam multimodal nonlinear-optical imaging of structurally complex events in cell-cycledynamics. J Physics: Photonics. (2021) 3:044001. doi: 10.1088/2515-7647/ac159a

[42] Chebotarev A Kelmanson I Ivanova A Khramova Y Katrukha V Kotova D . Multiphoton tools for hydrogen peroxide imaging *in vivo* with subcellular resolution. Sensors Actuators B: Chem. (2024) 410:135646. doi: 10.1016/j.snb.2024.135646

[43] Stoltzfus CR Barnett LM Drobizhev M Wicks G Mikhaylov A Hughes TE . Two-photon directed evolution of green fluorescent proteins. Sci Rep. (2015) 5:11968. doi: 10.1038/srep11968, PMID: 26145791 PMC4491718

[44] Drobizhev M Molina RS Callis PR Scott JN Lambert GG Salih A . Local electric field controls fluorescence quantum yield of red and far-red fluorescent proteins. Front Mol Biosci. (2021) 8:633217. doi: 10.3389/fmolb.2021.633217, PMID: 33763453 PMC7983054

[45] Niewerth D Kaspers GJ Assaraf YG van Meerloo J Kirk CJ Anderl J . Interferon-γ-induced upregulation of immunoproteasome subunit assembly overcomes bortezomib resistance in human hematological cell lines. J Hematol Oncol. (2014) 7:7. doi: 10.1186/1756-8722-7-7, PMID: 24418325 PMC3896789

[46] Russo G Landi R Pezone A Morano A Zuchegna C Romano A . DNA damage and Repair Modify DNA methylation and Chromatin Domain of the Targeted Locus: Mechanism of allele methylation polymorphism. Sci Rep. (2016) 6:33222. doi: 10.1038/srep33222, PMID: 27629060 PMC5024116

[47] Larson A . Multiphoton microscopy. Nat Photon. (2011) 5:1. doi: 10.1038/nphoton.an.2010.2

[48] Tu H Liu Y Turchinovich D Marjanovic M Lyngsø J Lægsgaard J . Stain-free histopathology by programmable supercontinuum pulses. Nat Photonics. (2016) 10:534–40. doi: 10.1038/nphoton.2016.94, PMID: 27668009 PMC5031149

[49] Xie N Zhang L Gao W Huang C Huber PE Zhou X . NAD+ metabolism: pathophysiologic mechanisms and therapeutic potential. Signal Transduct Target Ther. (2020) 5:227. doi: 10.1038/s41392-020-00311-7, PMID: 33028824 PMC7539288

[50] Brown M Driscoll J Monaco J . Structural and serological similarity of MHC-linked LMP and proteasome (multicatalytic proteinase) complexes. Nature. (1991) 353:355–7. doi: 10.1038/353355a0, PMID: 1922341

[51] Inholz K Anderl JL Klawitter M Goebel H Maurits E Kirk CJ . Proteasome composition in immune cells implies special immune-cell-specific immunoproteasome function. Eur J Immunol. (2024) 54:e2350613. doi: 10.1002/eji.202350613, PMID: 38458995

[52] Kincaid EZ Che JW York I Escobar H Reyes-Vargas E Delgado JC . Mice completely lacking immunoproteasomes show major changes in antigen presentation. Nat Immunol. (2011) 13:129–35. doi: 10.1038/ni.2203, PMID: 22197977 PMC3262888

[53] Ferrington DA Gregerson DS . Immunoproteasomes: structure, function, and antigen presentation. Prog Mol Biol Transl Sci. (2012) 109:75–112. doi: 10.1016/B978-0-12-397863-9.00003-1, PMID: 22727420 PMC4405001

[54] Moebius J Van Den Broek M Groettrup M Basler M . Immunoproteasomes are essential for survival and expansion of T cells in virus-infected mice. Eur J Immunol. (2010) 40:3439–49. doi: 10.1002/eji.201040620, PMID: 21108466

[55] Vachharajani N Joeris T Luu M Hartmann S Pautz S Jenike E . Prevention of colitis-associated cancer by selective targeting of immunoproteasome subunit LMP7. Oncotarget. (2017) 8:50447–59. doi: 10.18632/oncotarget.14579, PMID: 28881574 PMC5584149

[56] Hussong SA Roehrich H Kapphahn RJ Maldonado M Pardue MT Ferrington DA . A novel role for the immunoproteasome in retinal function. Invest Ophthalmol Vis Sci. (2011) 52:714–23. doi: 10.1167/iovs.10-6032, PMID: 20881299 PMC3053103

[57] Atkinson SP Collin J Irina N Anyfantis G Kyung BK Lako M . A putative role for the immunoproteasome in the maintenance of pluripotency in human embryonic stem cells. Stem Cells. (2012) 30:1373–84. doi: 10.1002/stem.1113, PMID: 22532526

[58] Cui Z Hwang SM Gomes AV . Identification of the immunoproteasome as a novel regulator of skeletal muscle differentiation. Mol Cell Biol. (2014) 34:96–109. doi: 10.1128/MCB.00622-13, PMID: 24164898 PMC3911280

[59] De Verteuil DA Rouette A Hardy MP Lavallee S Trofimov A Gaucher E . Immunoproteasomes shape the transcriptome and regulate the function of dendritic cells. J Immunol. (2014) 193:1121–32. doi: 10.4049/jimmunol.1400871, PMID: 24958905

[60] Dahlmann B . Mammalian proteasome subtypes: Their diversity in structure and function. Arch Biochem Biophys. (2016) 591:132–40. doi: 10.1016/j.abb.2015.12.012, PMID: 26724758

[61] Chen B Zhu H Yang B Cao J . The dichotomous role of immunoproteasome in cancer: Friend or foe? Acta Pharm Sin B. (2023) 13:1976–89. doi: 10.1016/j.apsb.2022.11.005, PMID: 37250147 PMC10213805

[62] Fricker LD . Proteasome inhibitor drugs. Annu Rev Pharmacol Toxicol. (2020) 60:457–76. doi: 10.1146/annurev-pharmtox-010919-023603, PMID: 31479618

[63] Leonardo-Sousa C Carvalho AN Guedes RA Fernandes PMP Aniceto N Salvador JAR . Revisiting proteasome inhibitors: molecular underpinnings of their development, mechanisms of resistance and strategies to overcome anti-cancer drug resistance. Molecules. (2022) 27:2201. doi: 10.3390/molecules27072201, PMID: 35408601 PMC9000344

[64] Roeten MSF Cloos J Jansen G . Positioning of proteasome inhibitors in therapy of solid Malignancies. Cancer Chemother Pharmacol. (2018) 81:227–43. doi: 10.1007/s00280-017-3489-0, PMID: 29184971 PMC5778165

[65] Stansborough RL Gibson RJ . Proteasome inhibitor-induced gastrointestinal toxicity. Curr Opin Support Palliat Care. (2017) 11:133–7. doi: 10.1097/SPC.0000000000000266, PMID: 28333868

[66] Basler M Dajee M Moll C Groettrup M Kirk CJ . Prevention of experimental colitis by a selective inhibitor of the immunoproteasome. J Immunol. (2010) 185:634–41. doi: 10.4049/jimmunol.0903182, PMID: 20525886

[67] Basler M Mundt S Muchamuel T Moll C Jiang J Groettrup M . Inhibition of the immunoproteasome ameliorates experimental autoimmune encephalomyelitis. EMBO Mol Med. (2014) 6:226–38. doi: 10.1002/emmm.201303543, PMID: 24399752 PMC3927957

[68] Ichikawa HT Conley T Muchamuel T Jiang J Lee S Owen T . Beneficial effect of novel proteasome inhibitors in murine lupus via dual inhibition of type I interferon and autoantibody-secreting cells. Arthritis Rheumatol. (2012) 64:493–503. doi: 10.1002/art.33333, PMID: 21905015 PMC4584406

[69] Turakhiya A Meyer SR Marincola G Böhm S Vanselow JT Schlosser A . ZFAND1 recruits p97 and the 26S proteasome to promote the clearance of arsenite-induced stress granules. Mol Cell. (2018) 70:906–919.e7. doi: 10.1016/j.molcel.2018.04.021, PMID: 29804830

[70] Enenkel C Kang RW Wilfling F Ernst OP . Intracellular localization of the proteasome in response to stress conditions. J Biol Chem. (2022) 298:102083. doi: 10.1016/j.jbc.2022.102083, PMID: 35636514 PMC9218506

[71] Tcherpakov M Delaunay A Toth J Kadoya T Petroski MD Ronai ZA . Regulation of endoplasmic reticulum-associated degradation by RNF5-dependent ubiquitination of JNK-associated membrane protein (JAMP). J Biol Chem. (2009) 284:12099–109. doi: 10.1074/jbc.M808222200, PMID: 19269966 PMC2673279

[72] Choi DS Choi DY Hong BS Jang SC Kim DK Lee J . Quantitative proteomics of extracellular vesicles derived from human primary and metastatic colorectal cancer cells. J Extracell Vesicles. (2012) 1:18704. doi: 10.3402/jev.v1i0.18704, PMID: 24009881 PMC3760640

[73] Rai A Greening DW Chen M Xu R Ji H Simpson RJ . Exosomes derived from human primary and metastatic colorectal cancer cells contribute to functional heterogeneity of activated fibroblasts by reprogramming their proteome. Proteomics. (2019) 19:e1800148. doi: 10.1002/pmic.201800148, PMID: 30582284

[74] Suwakulsiri W Rai A Xu R Chen M Greening DW Simpson RJ . Proteomic profiling reveals key cancer progression modulators in shed microvesicles released from isogenic human primary and metastatic colorectal cancer cell lines. Biochim Biophys Acta Proteins Proteom. (2019) 1867:140171. doi: 10.1016/j.bbapap.2018.11.008, PMID: 30502510

[75] Brealey J Lees R Tempest R Law A Guarnerio S Maani R . Shining a light on fluorescent EV dyes: Evaluating efficacy, specificity and suitability by nano-flow cytometry. J Extracell Biol. (2024) 3:e70006. doi: 10.1002/jex2.70006, PMID: 39399294 PMC11465455

[76] Popēna I Ābols A Saulīte L Pleiko K Zandberga E Jēkabsons K . Effect of colorectal cancer-derived extracellular vesicles on the immunophenotype and cytokine secretion profile of monocytes and macrophages. Cell Commun Signal. (2018) 16:17. doi: 10.1186/s12964-018-0229-y, PMID: 29690889 PMC5937830

[77] Ben-Nissan G Katzir N Füzesi-Levi MG Sharon M . Biology of the extracellular proteasome. Biomolecules. (2022) 12:619. doi: 10.3390/biom12050619, PMID: 35625547 PMC9139032

[78] Jia X Chen J Megger DA Zhang X Kozlowski M Zhang L . Label-free proteomic analysis of exosomes derived from inducible hepatitis B virus-replicating hepAD38 cell line. Mol Cell Proteomics. (2017) 16:S144–60. doi: 10.1074/mcp.M116.063503, PMID: 28242843 PMC5393393

[79] Zhu Y Chen X Pan Q Wang Y Su S Jiang C . A comprehensive proteomics analysis reveals a secretory path- and status-dependent signature of exosomes released from tumor-associated macrophages. J Proteome Res. (2015) 14:4319–31. doi: 10.1021/acs.jproteome.5b00770, PMID: 26312558

[80] Lai RC Tan SS The BJ Sze SK Arslan F de Kleijn DP . Proteolytic potential of the MSC exosome proteome: implications for an exosome-mediated delivery of therapeutic proteasome. Int J Proteomics. (2012) 2012:971907. doi: 10.1155/2012/971907, PMID: 22852084 PMC3407643

[81] Kulichkova V Artamonova T Lyublinskaya O Khodorkovskii M Tomilin A Tsimokha A . Proteomic analysis of affinity-purified extracellular proteasomes reveals exclusively 20S complexes. Oncotarget. (2017) 8:102134–49. doi: 10.18632/oncotarget.22230, PMID: 29254231 PMC5731941

[82] Kim J Zhao Y Kim HY Kim S Jiang Y Lee MJ . Extracellular vesicle-mediated delivery of 20S proteasomes enhances tau degradation in recipient cells. J Extracell Vesicles. (2025) 14:e70086. doi: 10.1002/jev2.70086, PMID: 40384174 PMC12086326

[83] Morozov V Morozov A Karpov VL . Functional 20S proteasomes in retroviruses: evidence in favor. Int J Mol Sci. (2024) 25:11710. doi: 10.3390/ijms252111710, PMID: 39519262 PMC11547158

[84] Woo MS Brand J Bal LC Moritz M Walkenhorst M Vieira V . The immunoproteasome disturbs neuronal metabolism and drives neurodegeneration in multiple sclerosis. Cell. (2025) 188:4567–4585.e32. doi: 10.1016/j.cell.2025.05.029, PMID: 40532699

[85] Guillaume B Stroobant V Bousquet-Dubouch MP Colau D Chapiro J Parmentier N . Analysis of the processing of seven human tumor antigens by intermediate proteasomes. J Immunol. (2012) 189:3538–47. doi: 10.4049/jimmunol.1103213, PMID: 22925930

[86] Khilji MS Verstappen D Dahlby T Burstein Prause MC Pihl C Bresson SE . The intermediate proteasome is constitutively expressed in pancreatic beta cells and upregulated by stimulatory, low concentrations of interleukin 1 β. PloS One. (2020) 15:e0222432. doi: 10.1371/journal.pone.0222432, PMID: 32053590 PMC7018053

[87] Watanabe A Yashiroda H Ishihara S Lo M Murata S . The molecular mechanisms governing the assembly of the immuno- and thymoproteasomes in the presence of constitutive proteasomes. Cells. (2022) 11:1580. doi: 10.3390/cells11091580, PMID: 35563886 PMC9105311

[88] Livneh I Cohen-Kaplan V Fabre B Abramovitch I Lulu C Nataraj NB . Regulation of nucleo-cytosolic 26S proteasome translocation by aromatic amino acids via mTOR is essential for cell survival under stress. Mol Cell. (2023) 83:3333–46. doi: 10.1016/j.molcel.2023.08.016, PMID: 37738964

[89] Fricke B Heink S Steffen J Kloetzel PM Krüger E . The proteasome maturation protein POMP facilitates major steps of 20S proteasome formation at the endoplasmic reticulum. EMBO Rep. (2007) 8:1170–5. doi: 10.1038/sj.embor.7401091, PMID: 17948026 PMC2267243

[90] Pack CG Yukii H Toh-e A Kudo T Tsuchiya H Kaiho A . Quantitative live-cell imaging reveals spatio-temporal dynamics and cytoplasmic assembly of the 26S proteasome. Nat Commun. (2014) 5:3396. doi: 10.1038/ncomms4396, PMID: 24598877

[91] Wu W Sahara K Hirayama S Zhao X Watanabe A Hamazaki J . PAC1-PAC2 proteasome assembly chaperone retains the core α4-α7 assembly intermediates in the cytoplasm. Genes Cells. (2018) 23:839–48. doi: 10.1111/gtc.12631, PMID: 30133132

[92] Joeris T Schmidt N Ermert D Krienke P Visekruna A Kuckelkorn U . The proteasome system in infection: impact of β5 and LMP7 on composition, maturation and quantity of active proteasome complexes. PloS One. (2012) 7:e39827. doi: 10.1371/journal.pone.0039827, PMID: 22768135 PMC3387237

