# Supplementary material for: Next-generation cell lines for profiling different proteasome forms and their implications in cancer
Source: Front Immunol. 2025 Dec 16;16:1672000. doi: 10.3389/fimmu.2025.1672000 (PMC12748260; doi:10.3389/fimmu.2025.1672000)
Supplement: Supplementary file 1 [file DataSheet1.docx]

**Supplementary material.**

**Supplementary Table 1.** The used primers.

| **Primer / Oligonucleotide** | **Sequence 5’ – 3’** |
| --- | --- |
| **gRNA cloning into pDG461** | |
| gRNA1_B5_olig_top | CACCTCAGCCACATTGTCACTGGAGACT |
| gRNA1_B5_olig_bottom | AAACAGTCTCCAGTGACAATGTGGCTGA |
| gRNA2_B5_olig_top | ACCGGTGGCTCTACCCCCTGAAAGAGGGGT |
| gRNA2_B5_olig_bottom | TAAAACCCCTCTTTCAGGGGGTAGAGCCAC |
| gRNA1_B10_olig_top | CACCGCACAGTTTCCTCCACTAGCTCC |
| gRNA1_B10_olig_bottom | AAACGGAGCTAGTGGAGGAAACTGTGC |
| gRNA2_B10_olig_top | ACCGTAAGCTGAGGCTTAGAGCTTGGAACAGT |
| gRNA2_B10_olig_bottom | TAAAACTGTTCCAAGCTCTAAGCCTCAGCTTA |
| **Donor fragments cloning into pAL-2T** |  |
| B5_Left_HA_fwd | CCGCCATGGCGGCCGCGGAGACATTACCTTCTCTGTAGGTTCTGGCTCTG |
| B5_Left_HA_rev | TCTCATGTAGATCAGCCACATTGTCACTGGAGACTCGTATCCAGCCATCC |
| B5_Right_HA_fwd | AAGAGGGTGAATGCAGCTGCTTGTGTTTC |
| B5_Right_HA_rev | CGACCTGCAGGTCGAATTCAGACATTGAGTAGTGAGTAGTGTAATGTTAACATCC |
| B5_Adapter_Olig_top | GACAATGTGGCTGATCTACATGAGAAGTATAGTGGCTCTACCCCCGGATCCGGAGGTGGAGGTTCAGGAG |
| B5_Adapter_Olig_bottom | CTCCTGAACCTCCACCTCCGGATCCGGGGGTAGAGCCACTATACTTCTCATGTAGATCAGCCACATTGTC |
| GFP_fwd | GGATCCGGAGGTGGAGGTTCAGGAGGTGGAGGTTCAGGTACCATGGTGAGCAAGGGCGAGGAGC |
| GFP_rev | CACAAGCAGCTGCATTCACCCTCTTTTACTTGTACAGCTCGTCCATGCC |
| B10_Left_HA_fwd | GCCGCCATGGCGGCCGCGGAGACATTGAGGTGAGAGCTGGAGATCGGGGAC |
| B10_Left_HA_rev | CACCTCCATAGCCTGCACAGTTTCCTCCACTAGCTCCAAGGTTAGTGGCT |
| B10_Right_HA_fwd | GCTGAGGCTTAGAGCTTGGAACAGGGGGGAATAAACCCAGAAAATAC |
| B10_Right_HA_rev | CGACCTGCAGGTCGAATTCAGACATGCTGGGGTTCTGGAGTTGTAGGTG |
| B10_Adapter_Olig_top | GGAAACTGTGCAGGCTATGGAGGTGGAGGGATCCGGAGGTGGAGGTTCAGGAGGTGGAGGTTCAGGTACC |
| B10_Adapter_Olig_bottom | GGTACCTGAACCTCCACCTCCTGAACCTCCACCTCCGGATCCCTCCACCTCCATAGCCTGCACAGTTTCC |
| PS_CFP_fwd | TTCAGGAGGTGGAGGTTCAGGTACCATGAGCAAGGGCGCCGAGCTG |
| PS_CFP_rev | CCTGTTCCAAGCTCTAAGCCTCAGCTTACTTGTACAGCTCATCCATGCC |
| **Control and sequencing** |  |
| B5_Left_Genome_Control_fwd (Primer A) | GAGGCAGGAGAATCACTTTAACCC |
| B5_Left_Genome_Control_rev (Primer B) | GAACTTGTGGCCGTTTACGTCG |
| B5_Right_Genome_Control_fwd (Primer C) | CGACCACTACCAGCAGAACACC |
| B5_Right_Genome_Control_rev (Primer D) | GCCCTGTGCATCTTTCCACCTC |
| B5_mRNA_UTR_fwd (Primer I) | CTGCCCACACTAGACATGGC |
| B5_mRNA_UTR_rev (Primer J) | GGGTCACTGTGTCCGTATTACC |
| B5_GFP_qPCR_fwd (Primer M) | CCACCTACAGAGATGCCTACTC |
| B5_GFP_qPCR_rev (Primer N) | GAACTTGTGGCCGTTTACGTC |
| B5_wt_qPCR_fwd  From Morozov et al. 2019 (Primer Q) | CTCCAAACTGCTTGCCAAC |
| B5_wt_qPCR_rev  From Morozov et al. 2019 (Primer R) | GTTCCCTTCACTGTCCACG |
| B10_Left_Genome_Control_fwd (Primer E) | GCAAGACCAGATTGGGTAAGTGGAC |
| B10_Left_Genome_Control_rev (Primer F) | AGCTTGCCGTAGGTGGCATC |
| B10_Right_Genome_Control_fwd (Primer G) | AACGAGAAGCGCGATCACATGATC |
| B10_Right_Genome_Control_rev (Primer H) | GTCTCTAGGAGGGGGTAGTACATGG |
| B10_mRNA_UTR_fwd (Primer K) | GAGGACTTTTTAGCTGCTCACTGGC |
| B10_mRNA_UTR_rev (Primer L) | CTGTATTTTCTGGGTTTATTCCCCCTTG |
| B10_CFP_qPCR_fwd (Primer O) | CCAGACAGTGAAGCCACTAACC |
| B10_CFP_qPCR_rev (Primer P) | TGGTGCAGATGAACTTCAGGG |
| B10_wt_qPCR_fwd  From Morozov et al. 2019 (Primer S) | GGTTCCAGCCGAACATGA |
| B10_wt_qPCR_rev  From Morozov et al. 2019 (Primer T) | ATGCGTCCACATTGCCC |
| B8_wt_qPCR_fwd From Burov et al. 2021 (Primer U) | GGTGAACAAGGTGATTGAG |
| B8_wt_qPCR_rev  From Burov et al. 2021 (Primer V) | GTTCTCCATTTCGCAGATAG |
| B8_mCherry_qPCR_fwd From Burov et al. 2021  (Primer W) | GAAGGAAGATGGTTGGGTGAAAG |
| B8_mCherry_qPCR_rev From Burov et al. 2021 (Primer X) | ACCTTGAAGCGCATGAACTCC |
| β-actin_qPCR_fwd  From Morozov et al. 2019 | TTGGCAATGAGCGGTTCC |
| β-actin_qPCR_rev  From Morozov et al. 2019 | GAGTTGAAGGTAGTTTCGTGG |
| SG_primer | TTTGTCTGCAGAATTGGC |
| SK_primer | TCTAGAACTAGTGGATC |
| M13_fwd | GTAAAACGACGGCCAGT |
| M13_rev | CAGGAAACAGCTATGAC |

| **Antibody** | **Manufacturer** | **RRID** |
| --- | --- | --- |
| Rabbit monoclonal antibodies to β2 | “Cell signaling, Boston, MA, USA” | AB_2798149 |
| Rabbit polyclonal antibodies to β5 | “GeneTex, Irvine, CA, USA” | AB_385014 |
| Rabbit monoclonal antibodies to β2i | “Abcam, Cambridge, UK” | AB_2895211 |
| Mouse monoclonal antibodies to GFP | “GeneTex, Irvine, CA, USA” | AB_2773724 |
| Rabbit monoclonal antibodies to GFP | “Cell signaling, Boston, MA, USA” | AB_1281301 |
| Rabbit monoclonal antibodies to mCherry | “Abcam, Cambridge, UK” | AB_2814891 |
| Mouse monoclonal antibodies to α subunits 1,2,3,5,6,7 | “ Enzo, Farmingdale, NY, USA” | AB_10541045 |
| Mouse monoclonal antibodies to Rpt6 | “Enzo, Farmingdale, NY, USA” | AB_10555017 |
| Mouse monoclonal antibodies to β - actin | “Cell signaling, Boston, MA, USA” | AB_2242334 |
| HRP-conjugated antibodies to Mouse IgG Heavy Chain | “ABclonal, Woburn, MA, USA” | AB_2864058 |
| HRP- conjugated antibodies to Rabbit IgG Heavy Chain | “GeneTex, Irvine, CA, USA” | *Cat. No. GTX628140-01 |
| HRP- conjugated antibodies to Rabbit IgG Light Chain | “ Cell signaling, Boston, MA, USA” | AB_2800208 |
| HRP- conjugated antibodies to Mouse IgG | “ Enzo, Farmingdale, NY, USA” | AB_10540652 |
| HRP- conjugated antibodies to Rabbit IgG | “Abcam, Cambridge, UK” | AB_10679899 |

**Supplementary Table 2.** The used antibodies.

**Supplementary figures.**

**
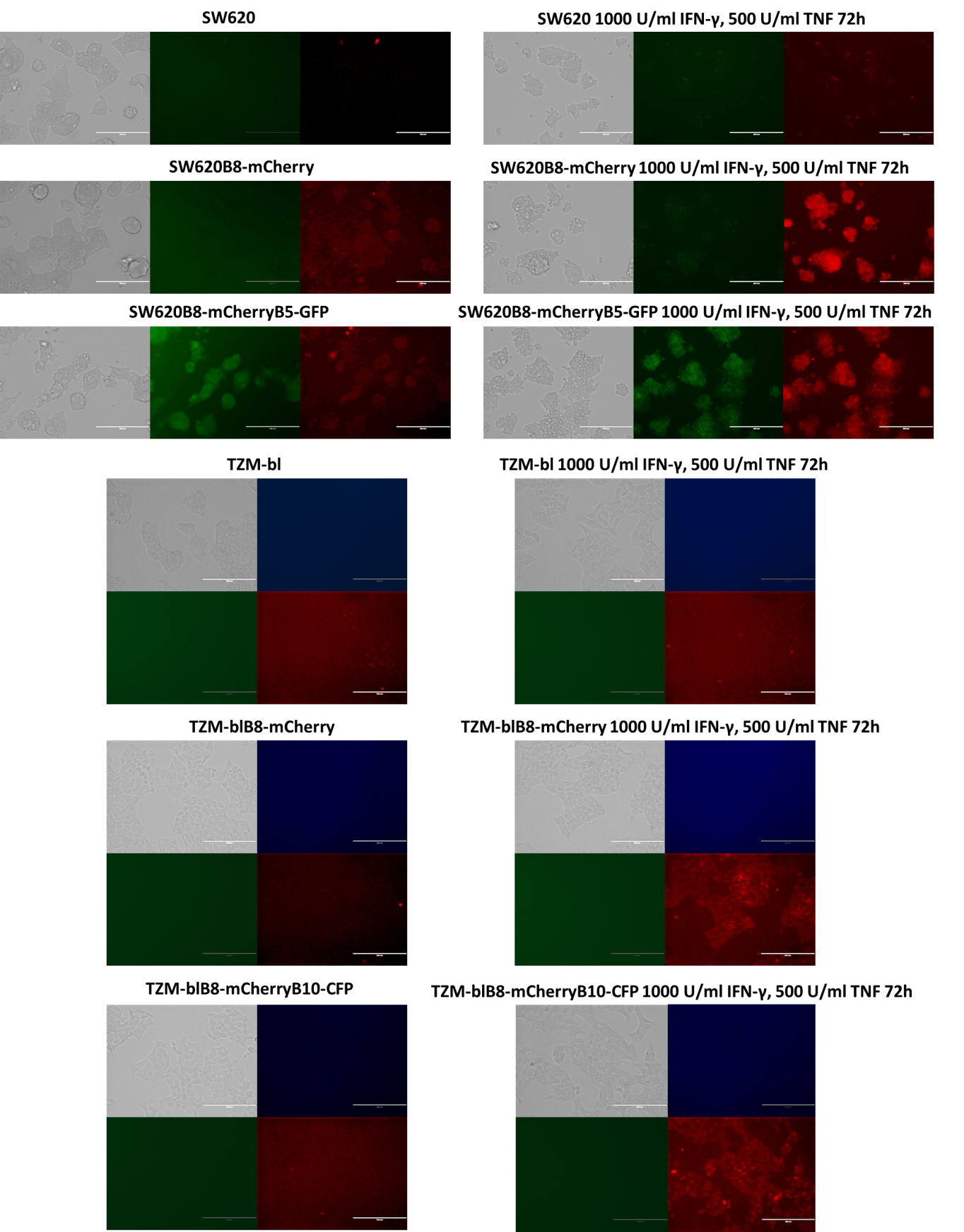
**

**Supplementary figure 1**. The fluorescent microscopy of control SW620, SW620B8-mCherry, SW620B8-mCherryB5-GFP, TZM-bl, TZM-blB8-mCherry, TZM-blB8-mCherryB10-CFP cells and cells treated with 1000 U/mL recombinant human IFN-γ and 500 U/mL recombinant human TNF for 72 h (both cytokines from R&D systems, Minneapolis, MN, USA). Scale bar – 200 µm.


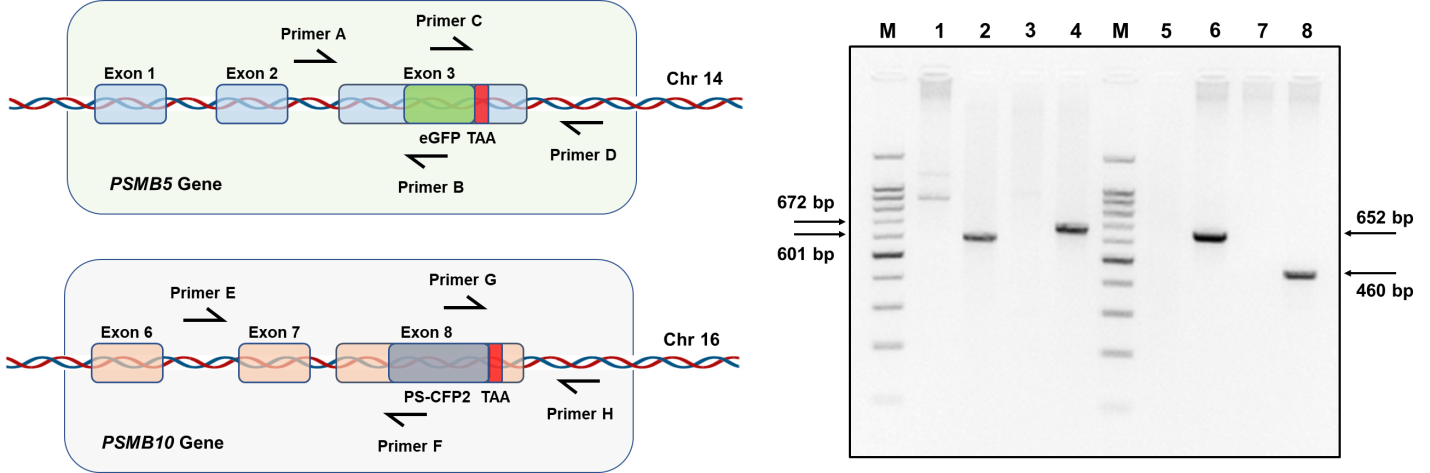


**Supplementary figure 2**. (Left) Positions of primers (Supplementary Table 1) that were used to amplify fragments from the genomic DNA of modified and control cells. (Right) Analysis of PCR results performed using the indicated primer pairs and genomic DNA from control and modified cells. Tracks 1 and 2 PCR products obtained with primers A – B and genomic DNA of SW620 (track 1) and SW620B8-mCherryB5-GFP (track 2) cells. Tracks 3 and 4 - PCR products obtained with primers C – D and genomic DNA of SW620 and SW620B8-mCherryB5-GFP cells, respectively. Tracks 5 and 6 - PCR products obtained with primers E – F and genomic DNA of TZM-bl (track 5) and TZM-blB8-mCherryB10-CFP cells (track 6). Tracks 7 and 8 - PCR products obtained with primers G – H and genomic DNA of TZM-bl and TZM-blB8-mCherryB10-CFP cells, correspondingly. M – 100+ bp DNA ladder (Evrogen, Moscow, Russia). The amplicons of the expected size (601 and 672 bp) and (652 and 460 bp) were observed in samples from SW620B8-mCherryB5-GFP and TZM-blB8-mCherryB10-CFP cells, respectively, but not in control cells.


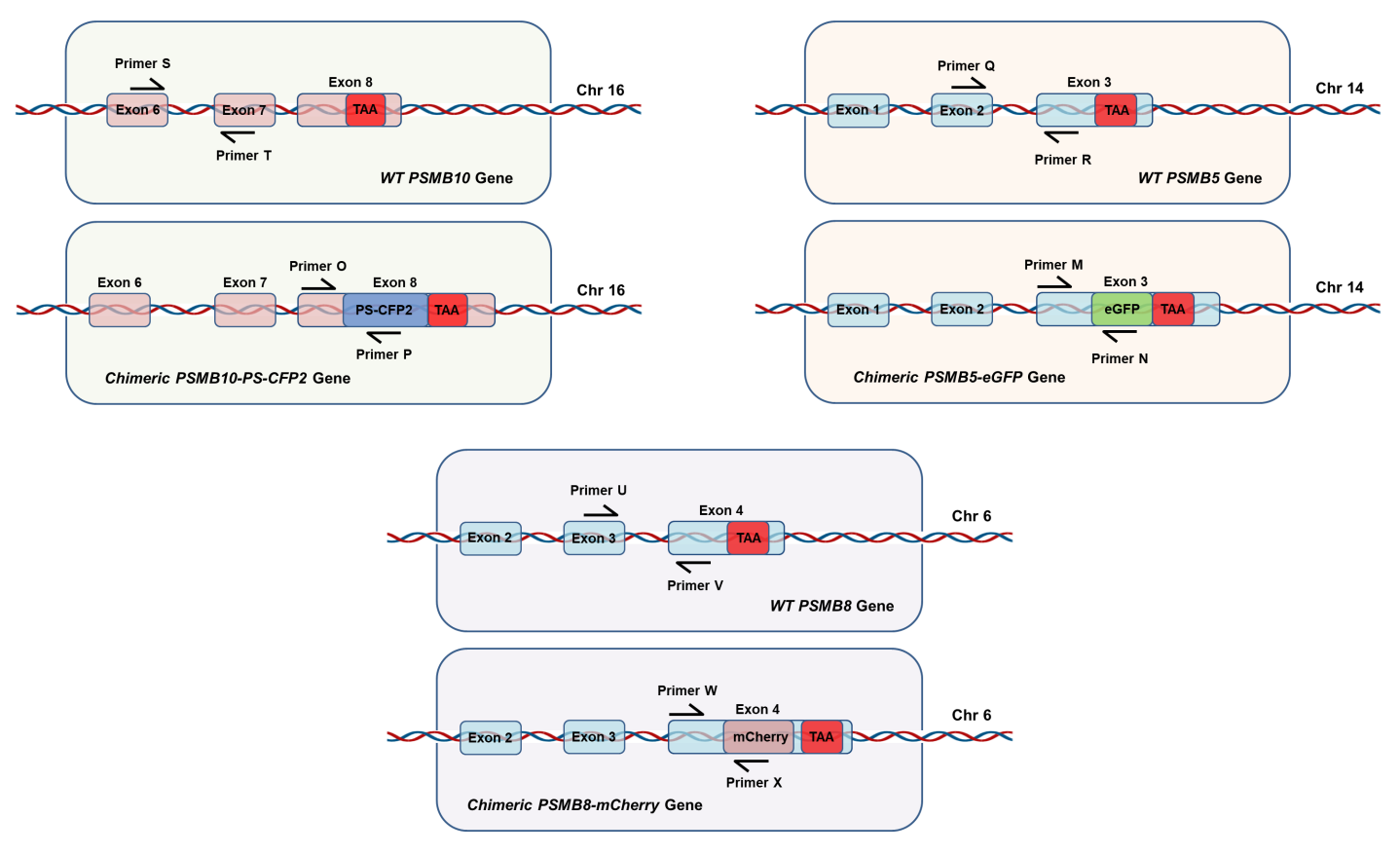


**Supplementary Figure 3**. Positions of primers (Supplementary Table 1) used to amplify fragments from cDNA samples obtained from control and modified cells.

**
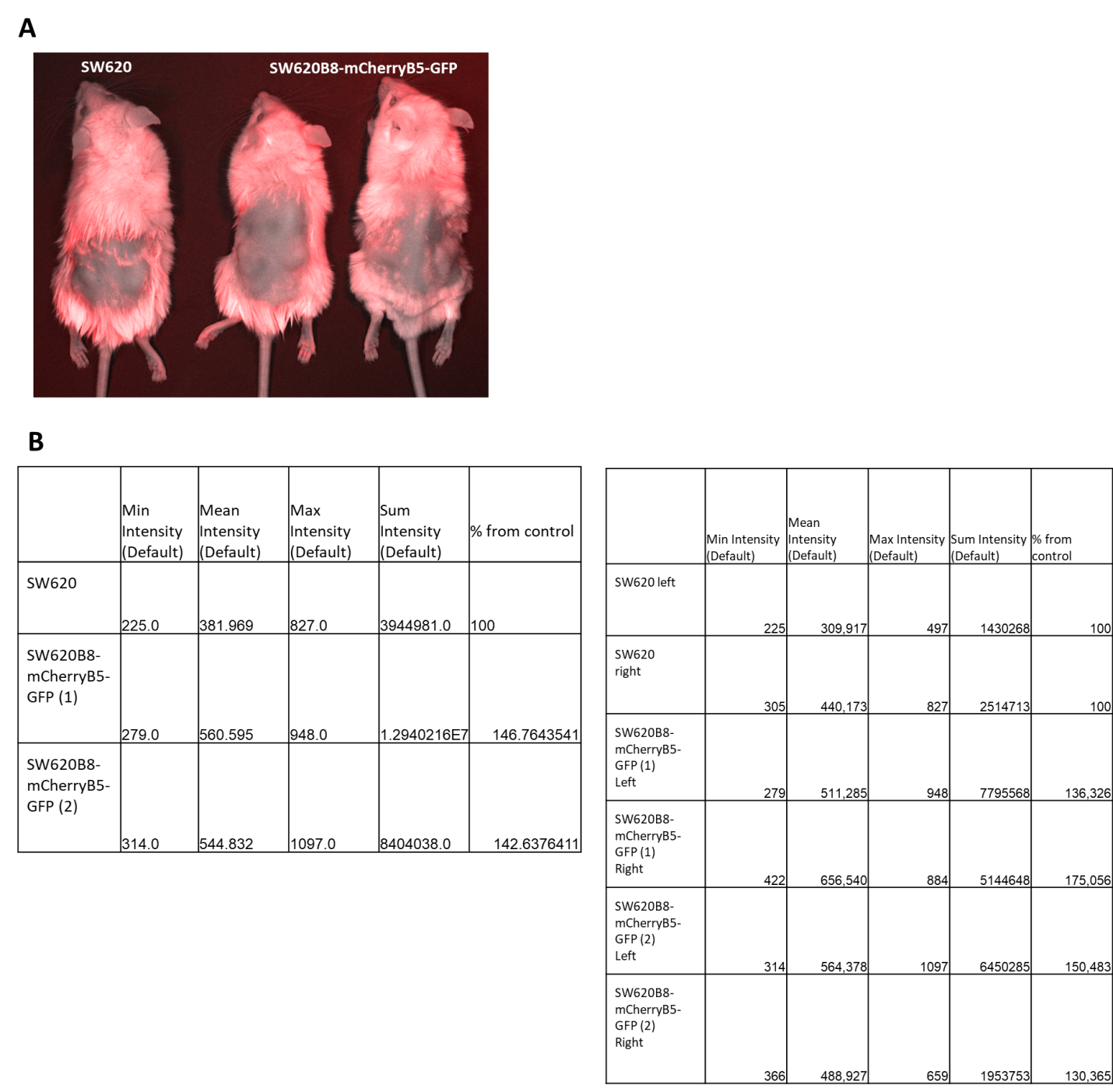
**

**Supplementary figure 4**. (**A**) The *in vivo* imaging of NSG-SGM3 mice subcutaneously injected with 300.000 of SW620 or SW620B8-mCherryB5-GFP cells. Mice are shown 14 days following tumor cell inoculation. (**B**) Quantification of mCherry fluorescence in tumors using the Icy software (Version 2.5.2.0).


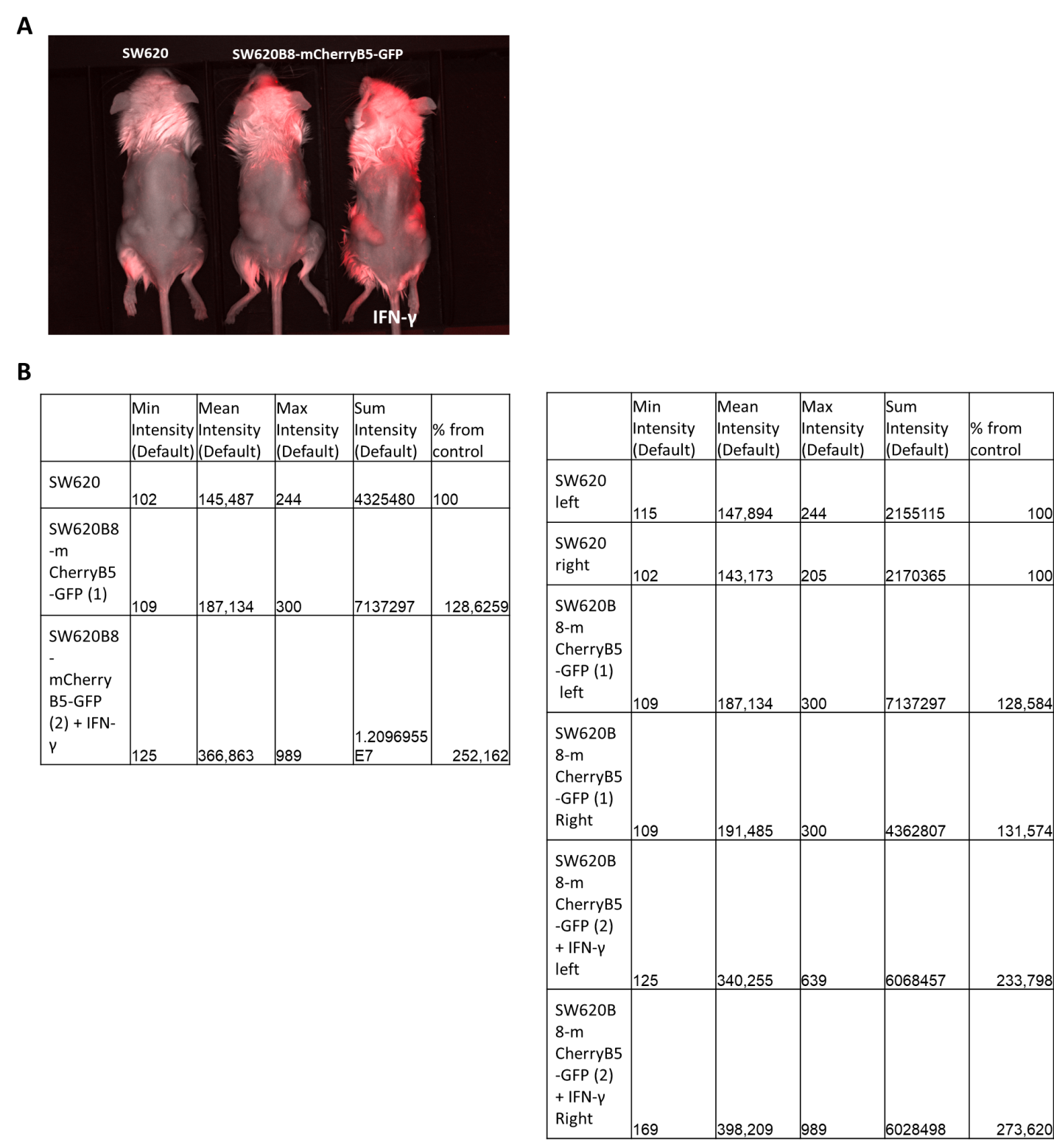


**Supplementary figure 5**. (**A**) The *in vivo* imaging of NSG-SGM3 mice subcutaneously injected with 300.000 of SW620 or SW620B8-mCherryB5-GFP cells. Mice are shown 17 days following tumor cell inoculation. The animal to the right received 50.000 U of recombinant human IFN-γ 72 h before examination. (**B**) Quantification of mCherry fluorescence in tumors using the Icy software (Version 2.5.2.0).

**
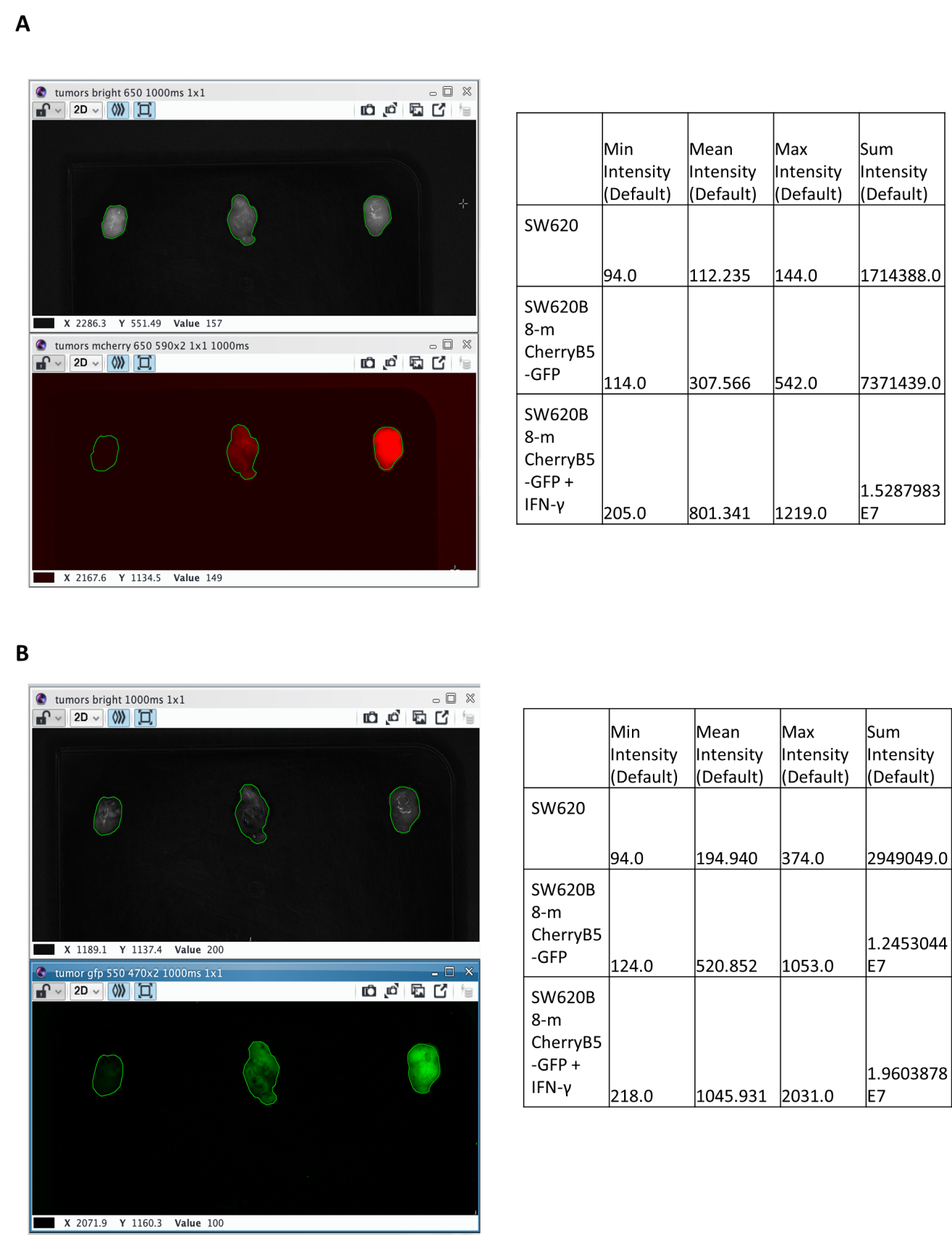
**

**Supplementary figure 6**. (**A**) The imaging of mCherry fluorescence in tumors obtained from NSG-SGM3 mice subcutaneously injected with 300.000 of SW620 or SW620B8-mCherryB5-GFP cells. The tumor to the right was obtained from the animal treated with 50.000 U of recombinant human IFN-γ for 72 h (left). Right, quantification of mCherry fluorescence in tumors using the Icy software (Version 2.5.2.0). (**B**) The imaging of EGFP fluorescence in tumors obtained from NSG-SGM3 mice subcutaneously injected with 300.000 of SW620 or SW620B8-mCherryB5-GFP cells. The tumor to the right was obtained from the animal treated with 50.000 U of recombinant human IFN-γ for 72 h (left). Right, quantification of EGFP fluorescence in tumors using the Icy software (Version 2.5.2.0).

**Supplementary movie 1**. Two-colour multiphoton imaging z-stack of the tumour derived from SW620B8-mCherryB5-GFP mouse treated with IFN-γ. Red channel – mCherry pumped by the 1045 nm laser, green channel – eGFP pumped by the 880 nm laser, blue channel – second harmonics pumped by the 1045 nm laser.

**Supplementary references**

1. Morozov, A.V., Burov, A.V., Astakhova, T.M. et al. (2019). Dynamics of the Functional Activity and Expression of Proteasome Subunits during Cellular Adaptation to Heat Shock. Mol Biol 53, 571–579. DOI:10.1134/S0026893319040071
2. Burov, A., Funikov, S., Vagapova, E., Dalina, A., Rezvykh, A., Shyrokova, E., et al. (2021). A Cell-Based Platform for the Investigation of Immunoproteasome Subunit β5i Expression and Biology of β5i-Containing Proteasomes. Cells. 10(11), 3049. DOI: 10.3390/cells10113049
